# Inflammation in the tumor-adjacent lung as a predictor of clinical outcome in lung adenocarcinoma

**DOI:** 10.1038/s41467-023-42327-x

**Published:** 2023-11-08

**Authors:** Igor Dolgalev, Hua Zhou, Nina Murrell, Hortense Le, Theodore Sakellaropoulos, Nicolas Coudray, Kelsey Zhu, Varshini Vasudevaraja, Anna Yeaton, Chandra Goparaju, Yonghua Li, Imran Sulaiman, Jun-Chieh J. Tsay, Peter Meyn, Hussein Mohamed, Iris Sydney, Tomoe Shiomi, Sitharam Ramaswami, Navneet Narula, Ruth Kulicke, Fred P. Davis, Nicolas Stransky, Gromoslaw A. Smolen, Wei-Yi Cheng, James Cai, Salman Punekar, Vamsidhar Velcheti, Daniel H. Sterman, J. T. Poirier, Ben Neel, Kwok-Kin Wong, Luis Chiriboga, Adriana Heguy, Thales Papagiannakopoulos, Bettina Nadorp, Matija Snuderl, Leopoldo N. Segal, Andre L. Moreira, Harvey I. Pass, Aristotelis Tsirigos

**Affiliations:** 1 Department of Pathology, NYU Grossman School of Medicine, New York, USA; 2 Applied Bioinformatics Laboratories, NYU Grossman School of Medicine, New York, USA; 3 Division of Precision Medicine, Department of Medicine, NYU Grossman School of Medicine, New York, USA; 4 Department of Cell Biology, NYU Grossman School of Medicine, New York, USA; 5 The Optical Profiling Platform at The Broad Institute of MIT And Harvard, Cambridge, USA; 6 Department of Cardiothoracic Surgery, NYU Grossman School of Medicine, New York, USA; 7 Division of Pulmonary, Critical Care and Sleep Medicine, NYU Grossman School of Medicine, New York, USA; 8 Genome Technology Center, Office of Science and Research, NYU Grossman School of Medicine, New York, USA; 9 Center for Biospecimen Research and Development, NYU Grossman School of Medicine, New York, USA; 10 Celsius Therapeutics, Cambridge, Massachusetts, USA; 11 Pharma Research & Early Development Informatics, Roche Innovation Center New York, New Jersey, USA; 12 Laura and Isaac Perlmutter Cancer Center, New York University Langone Health, New York, NY USA

**Keywords:** Non-small-cell lung cancer, Predictive medicine

## Abstract

Approximately 30% of early-stage lung adenocarcinoma patients present with disease progression after successful surgical resection. Despite efforts of mapping the genetic landscape, there has been limited success in discovering predictive biomarkers of disease outcomes. Here we performed a systematic multi-omic assessment of 143 tumors and matched tumor-adjacent, histologically-normal lung tissue with long-term patient follow-up. Through histologic, mutational, and transcriptomic profiling of tumor and adjacent-normal tissue, we identified an inflammatory gene signature in tumor-adjacent tissue as the strongest clinical predictor of disease progression. Single-cell transcriptomic analysis demonstrated the progression-associated inflammatory signature was expressed in both immune and non-immune cells, and cell type-specific profiling in monocytes further improved outcome predictions. Additional analyses of tumor-adjacent transcriptomic data from The Cancer Genome Atlas validated the association of the inflammatory signature with worse outcomes across cancers. Collectively, our study suggests that molecular profiling of tumor-adjacent tissue can identify patients at high risk for disease progression.

## Abstract (web summary)

Lung adenocarcinoma is often curable when diagnosed at an early stage, but a subsection of patients will progress. Here, the authors use multi-omics profiling to show that gene expression data can predict clinical outcome.

## Introduction

Despite advances in diagnosis and treatment, lung adenocarcinoma (LUAD), the most prevalent non-small cell cancer, remains the deadliest cancer in the United States. The risk of disease progression for early non-small cell lung cancer patients is currently about 30% after surgery^1^. With the emergence of improved treatments, recent studies have focused on creating predictive models for progression-free survival (PFS) and overall survival (OS) in lung cancer based on histology, mutations, gene expression, proteomics, and microbiome. Several studies have analyzed correlations between prognosis in resected early stage LUAD patients and histopathological patterns such as histological grading, predominant and high-grade patterns^2, 3^, and quantitative morphological features from histopathological images extracted with machine learning algorithms^4, 5^. However, studies integrating histology lack validation in clinical settings. Gene mutations in *SMARCA4* and *TP53*^6^, in *ATR*, *ERBB3*, *KDR*, and *MUC6*^7^, and fusions in *GOPC-ROS1* and *NTRK1-SH2DA*^7^ have also been identified as potential biomarkers for early stage LUAD recurrence after surgical resection, in contrast with *EGFR* which does not impact survival in early stages^8^. Nevertheless, these mutation prognostic tools need to be tested on independent external datasets. Gene expression is currently a growing field for the discovery of clinically relevant biomarkers for lung cancer recurrence prediction. Diverse machine learning algorithms integrated gene expression signatures and gene-expression based molecular subtypes, and selected key genes to elaborate prognostic models for lung cancer^9, 10^. However, these studies lack clinical reproducibility. Proteomics biomarkers have also been the center of many current studies. Models integrating proteins with distinct proteomic changes^11^, or incorporating a proteomics score^12^ were correlated to survival in NSCLC although they need to be validated on independent large-scale datasets as well. The emphasis thus far in studies attempting to stratify early-stage lung cancer has concentrated on signatures from the tumor itself. In this study, we hypothesized that tumor-adjacent normal lung tissue may hold significant prognostic information in early-stage lung cancer. Although a few studies suggested that airway transcriptomic profiles could add value to bronchoscopy for the diagnosis of the indeterminate pulmonary nodule without a direct biopsy of the tumor^13–20^, a review of the literature reveals that there have been no studies for the prognostication of lung cancer which investigate the transcriptome of lower airway samples, specifically using matched lung tissue from lung cancer bearing individuals. Seike et al. used a cytokine panel in both tumor and matched tumor-adjacent normal (TAN) tissue, but their TAN signature was only associated with lymph node metastasis and not with survival^21^. To investigate whether the transcriptome of the tumor-adjacent normal tissue can predict disease progression, we designed a matched tumor-normal study of early-stage lung adenocarcinoma patients with extensive follow-up. We used DNA-sequencing and RNA-sequencing to map the mutational and transcriptomic landscape respectively in this cohort in both the tumor and the tumor-adjacent normal. Our data shows that the transcriptome obtained from normal lung tissue, rather than that of the tumor, is the best predictor of progression. Furthermore, using unsupervised clustering for the de novo unbiased discovery of co-expressed gene modules, we identified a gene module characterized by TNF-α, NFκΒ and IL-17 signaling which is uniquely activated in the tumor-adjacent normal tissue of patients that eventually progress. We show that a simple inflammatory score derived without supervised training and/or a complicated set of parameters can effectively stratify patients by risk of disease progression. Using public datasets from TCGA, we show that the same inflammatory score can stratify patients in other cancer types. Finally, using single-nucleus RNA-sequencing on a subset of samples from our cohort, we discovered the cell types that are the main source of the prognostic inflammatory signature.

## Results

### A matched tumor-normal lung study: design and cohort characteristics

**Fig. 1:**
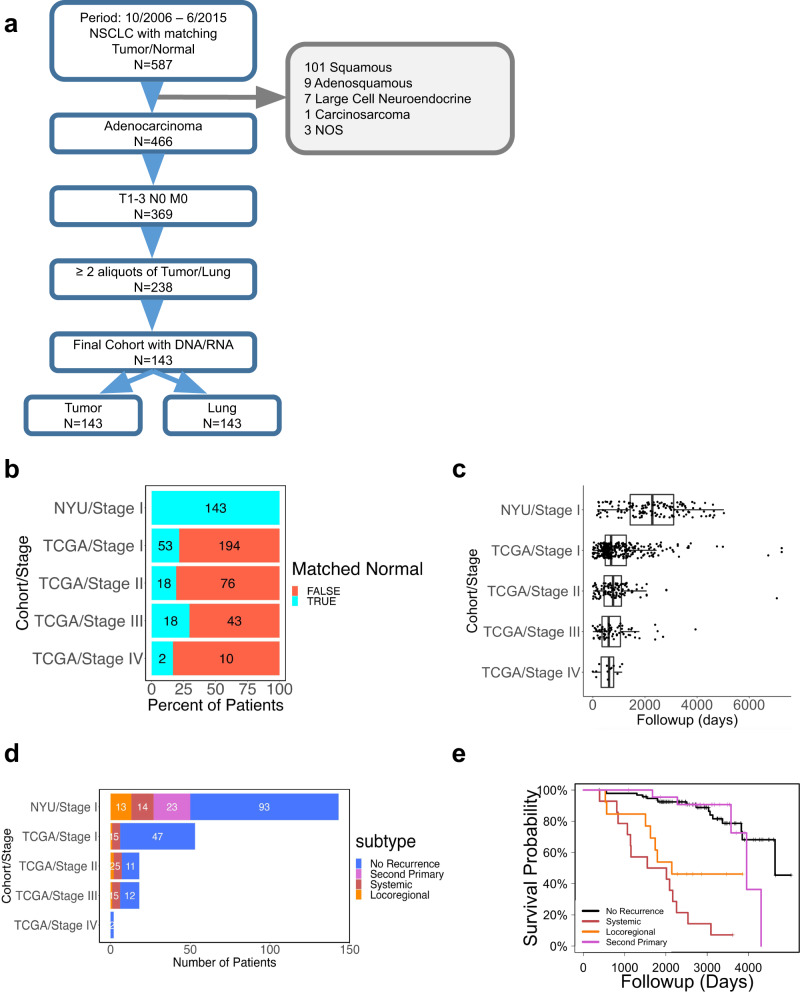
Study design and cohort characteristics. **a** CONSORT diagram. **b** Availability of matched tumor-adjacent normal lung patient samples in the NYU and TCGA cohorts. **c** Patient follow-up distribution in NYU Stage I (*n* = 145) cohort and in stage-specific TCGA cohorts (Stage I: *n* = 300, Stage II: *n* = 112, Stage III: *n* = 79, Stage IV: *n* = 14). Boxplots show medians (horizontal line in each box), interquartile ranges (boxes), 1.5 interquartile (whiskers) and each point represents a patient. **d** Number of patients with available matched normal lung samples by progression type across the NYU and TCGA cohorts. **e** Overall survival (OS) of patients with recurrence (systemic, locoregional) or second primary tumors.

In this study, we used a treatment-naive stage I lung adenocarcinoma cohort of patients with matched tumor and tumor-adjacent histologically normal lung samples (within the same lobe, segment, or wedge resection) obtained from our biorepository of prospectively collected specimens (see Methods). Patients included in the study at no time prior to surgery ever received any treatment for cancer (i.e. radiation, immunotherapy, or chemotherapy). A total of 143 patients matched our inclusion and exclusion criteria (Fig. 1a). To our knowledge, this is the largest study of matched tumor-normal early-stage cancer, as TCGA is limited to only 53 stage I patients with matched tumor-normal samples (Fig. 1b). Detailed information about our cohort and a comparison with the TCGA stage I cohort can be found in Supplementary Data 1. Overall, there were no major differences between the two cohorts in terms of patient characteristics, although our cohort consisted of slightly older patients (Supplementary Fig. 1a) with a lower median of pack years (Supplementary Fig. 1b).

Importantly, our cohort has an extensive follow-up, while the follow-up time in TCGA is rather limited (median follow-up of 2,284 days versus 701 days) (Fig. 1c). Substantially longer follow-up allows us to observe a significant number of disease progression events and enable the discovery of molecular signatures of progression-free survival. To date, we have recorded 50 patients (35%) in our cohort with disease progression. Specifically, we have identified 23 patients who developed a second primary tumor in the lung (see Methods for details), 13 patients have been diagnosed with locoregional recurrence in lymph nodes or tumor bed, and 14 with systemic metastasis in the brain, bone, pleura, liver, or adrenal gland; by comparison, only 6 patients have been documented with progressed disease in the TCGA stage I cohort (Fig. 1d). Distributions of age, smoking, sex, histologic and International Association for the Study of Lung Cancer (IASLC) grade in the progression and no progression groups are shown in Supplementary Fig. 1c–g. Univariate Cox regression analysis recapitulated previous results (Supplementary Fig. 2). The overall survival for patients with systemic or locoregional recurrence is worse than in patients with a second primary tumor (Fig. 1e).

### Mutational and transcriptomic profiling of matched tumor-normal lung specimens

We first performed DNA sequencing of the patient samples using the NYU GenomePACT panel which covers the exons of 580 protein-coding genes plus the TERT promoter (see Methods for details). For each patient, we used samples from the tumor, tumor-adjacent normal (TAN) lung and normal blood (see Supplementary Data 2 for quality assessment). We then performed RNA-seq on all 286 samples (143 tumors and 143 tumor-adjacent normal lungs). The RNA-seq analysis generated adequate sequenced reads and high percentages of uniquely aligned reads for the majority of samples (Supplementary Data 3): 15 tumor and 10 normal lung samples were excluded from downstream analysis due to low library quality. Eventually, 123 matched tumor-normal samples (86% of the initial set of 143 matched samples) were deemed high-quality RNA-seq samples and used for the downstream analyses. As expected, Principal Component Analysis (PCA) of the RNA-seq data shows a separation of tumor and normal samples (Supplementary Figure 3a). Supplementary Fig. 3b summarizes the workflow of sequencing and quality control. In summary, the vast majority of the patients in our cohort successfully underwent mutational and transcriptional profiling using a total of 5 samples per patient. To ensure that each set of 5 samples indeed belongs to the same patient and exclude possibility of sample swapping and/or mislabeling during sample collection, library preparation or sequencing, we performed a relatedness analysis based on common variants (see Methods for details). The full results of the genotyping analysis are included in Supplementary Fig. 4 and demonstrate that the different samples were all properly labeled.

### Mutations are poor predictors of clinical outcome in early-stage lung adenocarcinoma

**Fig. 2:**
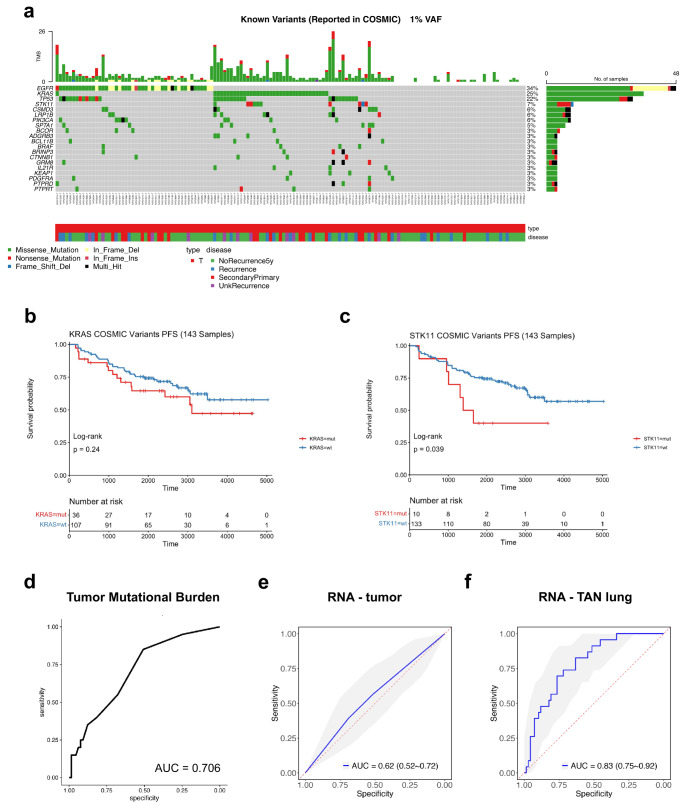
Multi-omic profiling of matched tumor-normal stage I lung adenocarcinomas. **a** Oncoprint of frequently mutated genes in the tumor samples (type T stands for tumor). **b** Kaplan–Meier progression-free survival (K-M PFS) plots comparing patients with and without *KRAS* mutation. **c** K-M PFS plots comparing patients with and without *STK11* mutation. **d** ROC curve and AUC of prediction of 5-year recurrence based on patient TMB values. **e** ROC curves of elastic net model built on top-200 highly variable genes in tumor to predict 5-year recurrence. 95% confidence intervals was also shown in gray. **f** ROC curves of elastic net model built on top-200 highly variable genes in tumor-adjacent normal (TAN) tissue to predict 5-year recurrence. 95% confidence intervals was also shown in gray.

Analysis of the DNA sequencing data obtained from the patients’ tumors revealed a mutational landscape with the typical distribution of frequently mutated genes in early-stage lung adenocarcinomas (LUAD): 34% *EGFR*, 25% *KRAS*, 22% *TP53* and 7% *STK11* (Fig. 2a). We then looked at genes that may be mutated at different rates in patients that progress compared to those that do not. We defined two groups, the progression group comprising all disease progression events regardless of the progression type and the no progression group comprising all patients that did not progress with at least 5-year follow-up. As expected, stratifying patients by *EGFR* mutational status does not yield a statistical difference in PFS, while even a stratification by *KRAS* or *STK11* mutational status is not significant (*p*-value > 0.01, Fig. 2b,c). The same was true for recurrence-free survival (RFS), with the only exception of *TP53* which was found to be significantly associated with recurrence (*p*-value = 0.0053, log-rank test). However, tumor mutational burden (TMB) was found to be a modest predictor of 5-year recurrence (AUC = 0.706) (Fig. 2d). Mutation calling in the tumor-adjacent normal samples (using blood as germline reference) showed mutations with a variant allele frequency (VAF) cutoff of 1% in 31 TAN samples with only three patients showing mutations present in the primary tumor. In addition, only three patients had a single mutation with a VAF higher than 5% (one patient with a *TP53* stop-gain mutation, one with a non-synonymous PRDM16 mutation and one with a non-synonymous DNMT3A mutation), suggesting that the presence of mutations in the TAN is rather limited (Supplementary Data 4). Combined, this data suggest that mutations are poor predictors of PFS in stage I LUAD.

### Gene expression in tumor-adjacent normal holds significant prognostic information

To identify better prognostic markers for early stage LUAD, we then tested whether gene expression obtained from bulk RNA-seq can provide prognostic information and predict 5-year recurrence. To this end, we constructed an elastic net machine learning model to predict systemic and locoregional recurrence, using nested cross-validation to allow for automatic, unbiased hyper-parameter optimization ensuring no data leakage from training to test sets (see “Methods” for details). We found that the transcriptomic signature in the tumor does not predict recurrence (AUC = 0.62, 95% confidence interval = [0.52–0.72]) (Fig. 2e) and cannot stratify the patients into high- and low-risk groups (PFS log-rank test *p*-value = 0.456). However, our analysis determined that a model based on transcriptomic information from the TAN samples shows superior performance (AUC = 0.83, 95% confidence interval = [0.75–0.92], see Fig. 2f) and is able to stratify the patients into high- and low-risk groups (PFS log-rank test *p*-value = 0.007), significantly outperforming the tumor-based model (Delong’s test, *p*-value = 0.0033). Highlighting the importance of including TAN samples in our study and suggesting that TAN lung tissue may contribute to recurrence. The TAN-based model can further identify patients with future systemic recurrence (Supplementary Fig. 5a; AUC = 0.85, 95% confidence interval = [0.76–0.94], sensitivity = 0.923, specificity = 0.676), and with future locoregional recurrence (Supplementary Figure 5b; AUC = 0.82, 95% confidence interval = [0.72–0.91], sensitivity = 1.000, specificity = 0.574). However, future second primaries are not accurately detected (Supplementary Fig. 5c; AUC = 0.70, 95% confidence interval = [0.60–0.79], sensitivity = 0.591, specificity = 0.809), possibly due to the different biology of secondary tumors that occur independently of the first primary tumor. In addition, we tested the supervised model on lung cancer cohorts from TCGA. Despite the limited TAN data on TCGA, the NYU model had a decent performance on the TCGA lung adenocarcinoma (LUAD) TAN transcriptome (AUC = 0.75, 95% confidence interval = [0.57, 0.89]). In fact, the model performed equally well when applied on the TAN transcriptome of the TCGA lung squamous cell carcinoma (LUSC) cohort (AUC = 0.74, 95% confidence interval = [0.47, 0.93]). Combining these two cohorts yielded a similar performance (AUC = 0.75, 95% confidence interval = [0.59, 0.88]). Of note, IASLC grade showed significantly lower performance in predicting progression (Supplementary Fig. 5d; AUC = 0.64, 95% confidence interval = [0.56–0.71]) or recurrence (Supplementary Fig. 5e; AUC = 0.74, 95% confidence interval = [0.65–0.82]). Thus, our data suggest superior power of TAN transcriptome-based models for prediction of PFS, systemic and locoregional recurrence in LUAD than tumor-based models.

### Co-expressed gene module analysis reveals the activation of inflammatory pathways in tumor-adjacent normal lung tissue

**Fig. 3:**
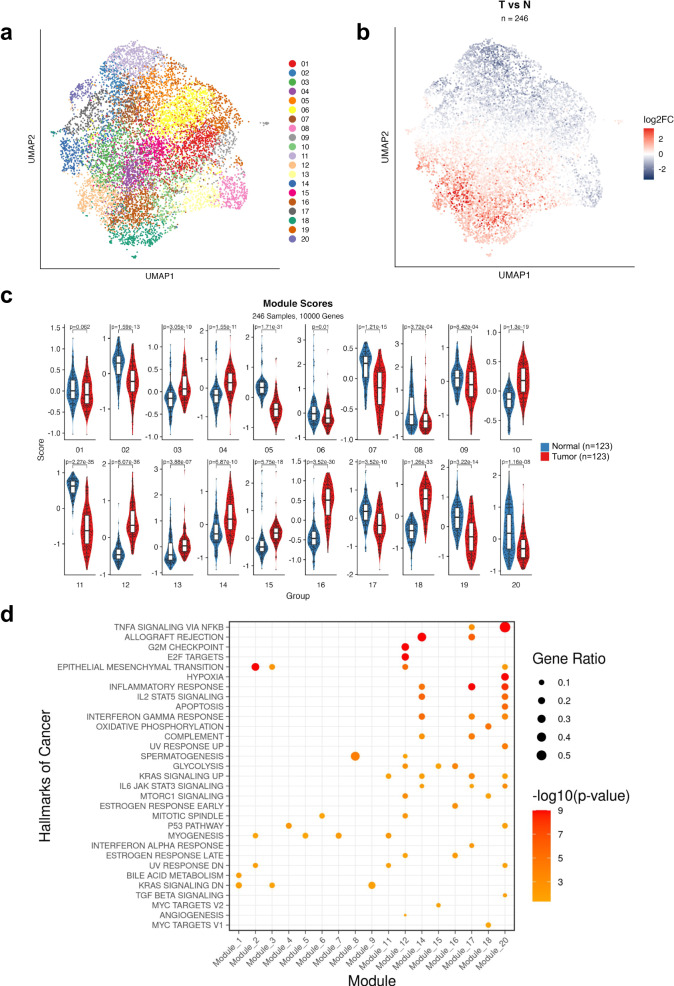
Gene co-expression modules in tumor and tumor-adjacent normal tissue. **a** UMAP representation of 20 gene co-expression modules—each point on the map corresponds to a gene. **b** UMAP representation annotated by log-fold change tumor vs TAN for each genes on the map. **c** Boxplots comparing modules scores in tumor and TAN samples in each module. Boxplots show medians (horizontal line in each box), interquartile ranges (boxes), 1.5 interquartile (whiskers) and each point represents a patient. The *p*-values are calculated using two-sided Wilcoxon rank sum test. **d** Dot plot of enriched hallmarks across modules (module 10, 13, and 19 have no highly significant associations). The *p*-values are calculated using Fisher’s exact test (one-tailed) and they are adjusted using False Discovery Rate (FDR).

To further understand the underlying transcriptional programs holding prognostic value in the TAN in comparison to the tumor tissue, we set out to characterize the transcriptional programs specifically activated in TAN. Instead of relying on complex supervised machine learning models (Fig. 2e, f) with a potentially large number of parameters and questionable capacity to generalize in a clinical setting, we decided to further analyze the 246 matched tumor-normal RNA-seq samples using an unsupervised unbiased approach. Briefly, we selected the top 10,000 most variable genes, scaled their expression across samples and performed dimensionality reduction using unifold manifold approximation and projection (UMAP; each point on the UMAP represents a gene, see Methods for details). Unsupervised clustering revealed 20 gene clusters, i.e., co-expressed gene modules, or, simply, modules (Fig. 3a). We then colored each gene by its log-fold change from TAN to tumor samples, revealing clusters of genes with higher expression in the tumor samples (red color) and clusters with higher expression in the normal samples (blue color) as shown in Fig. 3b. To identify the modules that have overall higher expression in tumor compared to tumor-adjacent normal and vice versa, we defined a score for each module as the average scaled gene expression of genes in the module (per patient, per tissue type). Indeed, we found that several modules have significantly higher average expression in the normal samples (modules 2, 5, 6, 7, 8, 9, 11, 17, 19, and 20), while others were more highly expressed in tumor samples (modules 3, 4, 10, 12, 13, 14, 15, 16, and 18) (Fig. 3c). We then characterized each module based on its association with hallmarks, gene sets with well-defined biological states or processes^22^. The module found to be associated with the highest number of hallmarks was module 20 (Fig. 3d). Strikingly, although module 20 has a higher score in the normal lung tissue compared to tumor, it was found to be significantly enriched in a large number of hallmarks that are typically linked to cancer, confirming that tumor-adjacent normal tissue is not entirely normal, in agreement with previous studies. In particular, inflammatory signaling pathways (TNF-α, IL-17, and NFκΒ), IL-2 and IL-6 signaling, interferon-gamma response and hypoxia were found to be highly enriched in module 20 genes. The full list of enriched hallmarks, KEGG pathways, and Gene Ontology (GO) terms and all statistics can be found in Supplementary Data 5.

### Transcriptomic signatures of lung adenocarcinoma progression in tumor and tumor-adjacent normal tissue

**Fig. 4:**
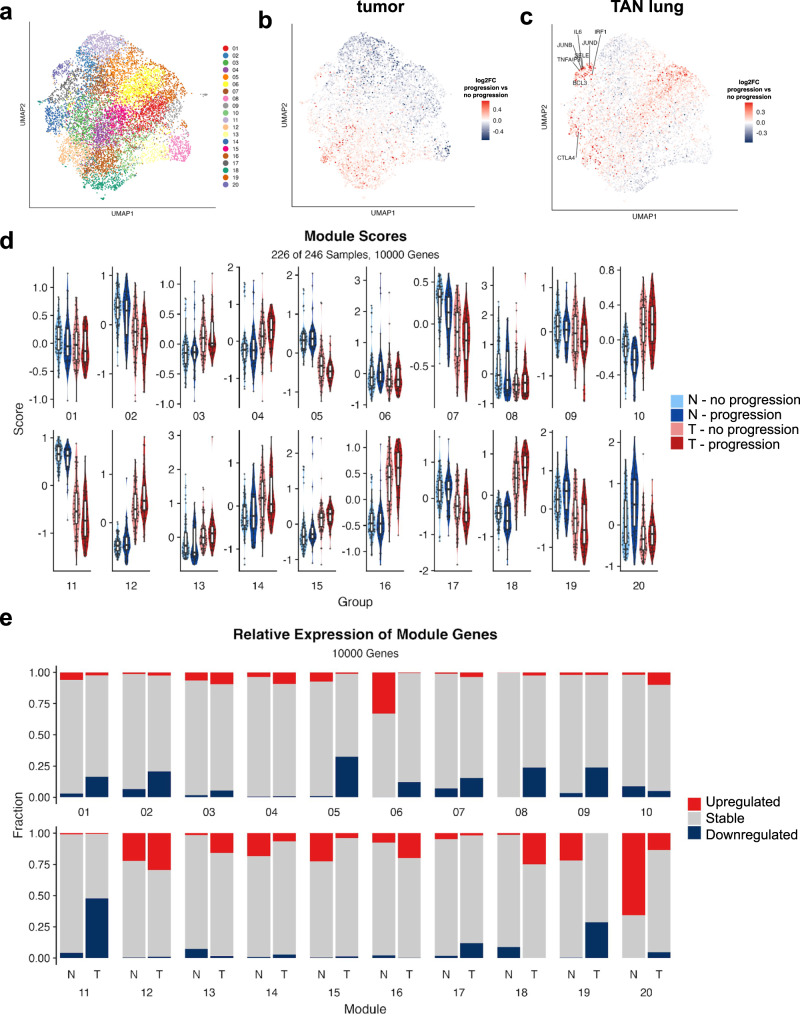
Gene co-expression modules in lung adenocarcinoma progression. **a** UMAP representation of 20 gene co-expression modules. **b** UMAP representation annotated by log-fold change progression (red color) vs no progression (blue color) in tumor samples. **c** UMAP representation annotated by log-fold change progression (red color) vs no progression (blue color) in normal samples. **d** Boxplots comparing modules scores by progression status in tumor (T) and normal (N) tissue in each module. Boxplots show medians (horizontal line in each box), interquartile ranges (boxes), 1.5 interquartile (whiskers) and each point represents a patient. **e** Percentages of up- and downregulated genes (progression vs no progression) in tumor (T) and tumor-adjacent normal (N) tissue in each module.

Motivated by the observation that inflammatory and other pathways linked to cancer are activated in TAN, we hypothesized that activation of such pathways and related gene modules, most notably module 20 which was found to be associated with the highest number of cancer-related hallmarks, may inform disease progression. To test this hypothesis, we identified genes that are differentially expressed in the tumor or TAN tissue between the group of patients that eventually progress and the ones that do not. More specifically, patients from our matched tumor-normal cohort were divided into two groups: the progression group comprised all patients with any type disease progression (*n* = 45), while the no progression group comprised all patients that have not progressed with at least 5 years of follow-up time (*n* = 68). Differential expression analysis between the two groups was performed separately on the tumors (Supplementary Fig. 6a) and the normal lung samples (Supplementary Fig. 6b). We observed a similar number of differentially expressed genes in the two tissue types (672 in tumor and 474 in TAN), while the two lists of differentially expressed genes showed minimal overlap, suggesting that the dysregulated pathways in patients that eventually progress are different in the tumor and the TAN tissue. The results of the differential expression analysis are available as Supplementary Data 6. We then explored the distribution of differentially expressed genes across the co-expressed gene modules. We colored each gene in the gene module UMAP (Fig. 4a) by the log-fold change in expression between the progression and no progression groups, separately for the tumor (Fig. 4b) and the TAN samples (Fig. 4c). Visual inspection and comparison of the UMAPs revealed that upregulated genes in patients that eventually progressed are localized almost exclusively in particular modules, especially in the TAN lung samples. The most prominent such module is module 20 which has a high percentage of upregulated genes in the TAN lung tissue of patients who progress. This is confirmed by module aggregate expression analysis (Fig. 4d, Supplementary Fig. 6c), calculating the percentages of up- and downregulated genes across modules in the two tissue types (Fig. 4e). Clearly, module 20 is highly biased towards upregulated genes in the progressors’ group in the TAN tissue, but not in the tumor. Thus, our data suggests an association of module 20 with TAN as well as progression.

### A multi-modal association map for refined patient classification

**Fig. 5:**
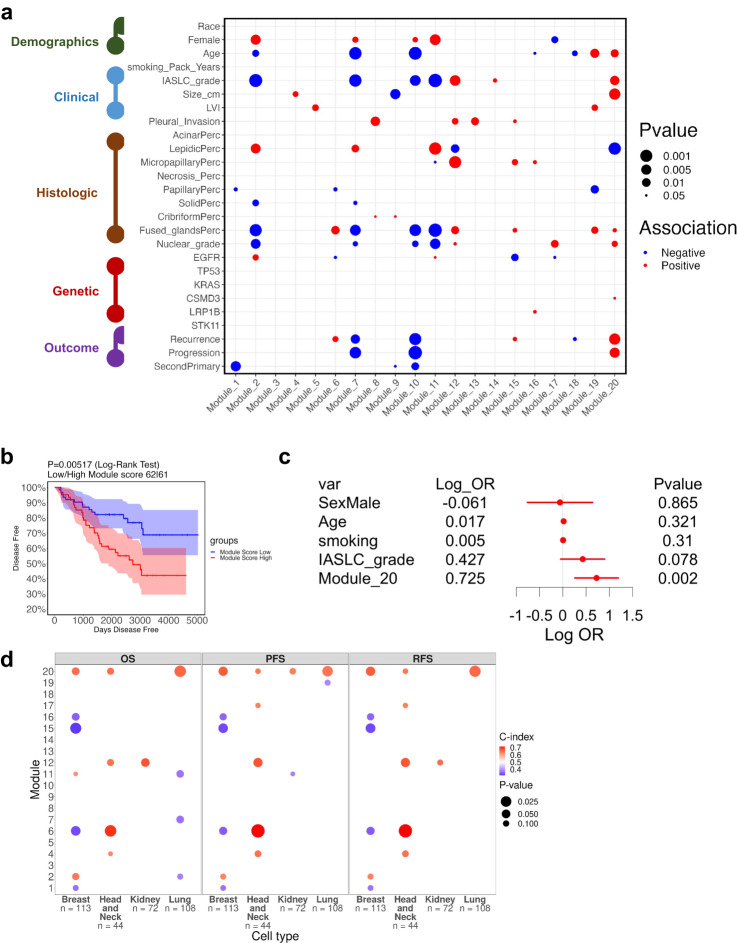
Association of module scores in tumor-adjacent normal tissue with different variables. **a** Positive and negative associations of demographic, clinical, histologic, genetic and outcomes with module scores in TAN tissue. Pearson and spearman correlation tests were done for continuous and categorical variables separately. **b** Kaplan–Meier progression-free survival curve for patients with high (*n* = 62) and low (*n* = 61) module 20 scores in TAN tissue. 95% confidence interval was also shown in shaded blue and red. **c** Multi-variate modeling of time-to-progression (*n* = 123), log of odds ratio and data are presented as mean values with 95% confidence intervals, *p*-values are calculated based on Wald test for each variable. **d** Dot plot of c-index values between module scores and outcome (overall-survival (OS), progression-free survival (PFS) and recurrence-free survival (RFS)) per module in TCGA cohorts grouped by tissue type (non-adjusted).

To further characterize the identified gene modules in the TAN, we performed a comprehensive association analysis of module scores with demographic, clinical, histologic, genetic, and survival data (Fig. 5a). The only module significantly associated with poor survival was module 20 (Fig. 5b) and it was found to be an independent predictor of clinical outcome in a multivariate analysis (Fig. 5c) with a log odds-ratio of 0.725 (*p*-value = 0.002). Intriguingly, IASLC grade which is part of the updated WHO guidelines of lung adenocarcinoma, was not found significant in the same multivariate analysis. The sensitivity of this model in predicting recurrence was found to be 0.821 with specificity at 0.491. The association map in Fig. 5a provides a wealth of information that can be used in future bigger studies to not only stratify patients into highly refined groups based on a combination of demographic, clinical, histologic, and genetic data, but also generate hypotheses regarding the underlying biological processes and pathways involved by integrating with transcriptomic data from the tumor and the tumor-adjacent normal. For example, modules 7 and 10 are associated with younger patients, are broadly associated with low grade tumors, absence of high-risk histologic patterns (solid and fused grands) and better outcomes. Modules 19 and 20 are associated with older patients and high-grade tumors, although only module 20 was found significantly associated with clinical outcome. Modules 8, 12, and 13 are associated with pleural invasion. Interestingly, none of the modules is associated with mutations, supporting our original hypothesis that the tumor-adjacent normal tissue may be a valuable source of biomarkers for progression, independent of the genetic makeup of the tumor itself. In particular, module 20 activation occurs in patients that progress independent of the driver mutation of their tumors. Next, we tested whether smaller gene subsets of module 20 hold equivalent prognostic information. To this end, we analyzed the top *n* = 10, 20, 40, … most highly expressed genes in module 20 (ranked by average expression). First, we calculated the correlation of the reduced module 20 signatures with the full signature, showing that even when using a very small number of genes the correlation remains significantly high (Supplementary Fig. 7a–b). Then, we tested whether the reduced signatures are still prognostic, and as expected from the observed high correlations, indeed, they remain prognostic in terms of PFS (Supplementary Fig. 7c) and RFS (Supplementary Fig. 7d). Finally, we derived the same type of association map for the tumor samples (Supplementary Fig. 8a). Module 20 in the tumor has no significant association with survival in univariate or multivariate analysis (Supplementary Fig. 8b, c).

### Testing the inflammatory module 20 signature on additional cancer types

To further test whether the module 20 inflammatory signature can be more broadly applied to the TAN tissue of other cancer types, we performed an analysis of the data obtained from normal tissue in TCGA. Given the limited number of TAN samples in TCGA with RNA-seq data, we were only able to find four primary tumor sites with at least 40 tumor-adjacent normal samples and at least two progression events across all stages: breast, lung, kidney, and head/neck cancer. We calculated c-index values between module scores and progression-free survival for each module and each cancer type (c-index values are higher when high module scores are associated with worse survival). The results of this analysis are shown in Fig. 5d, demonstrating that module 20 is the only module score that is consistently and significantly associated with poor outcome in all four cancer types. Taken together, these findings suggest a prominent role of module 20 in progression. As shown above in Fig. 3d, this module is enriched in inflammatory signaling pathways (TNF-α, IL-17, and NFκΒ) and hallmarks of cancer (IL-2 and IL-6 signaling, interferon-gamma response and hypoxia), even though it is a module that is more highly expressed in the adjacent normal compared to the actual tumor. This observation suggests that patients who eventually progress, have compromised lungs bearing hallmarks of disease progression that are not necessarily observable in the adjacent tumors.

### Profiling the tumor and tumor-adjacent normal tissue at single-cell resolution

**Fig. 6:**
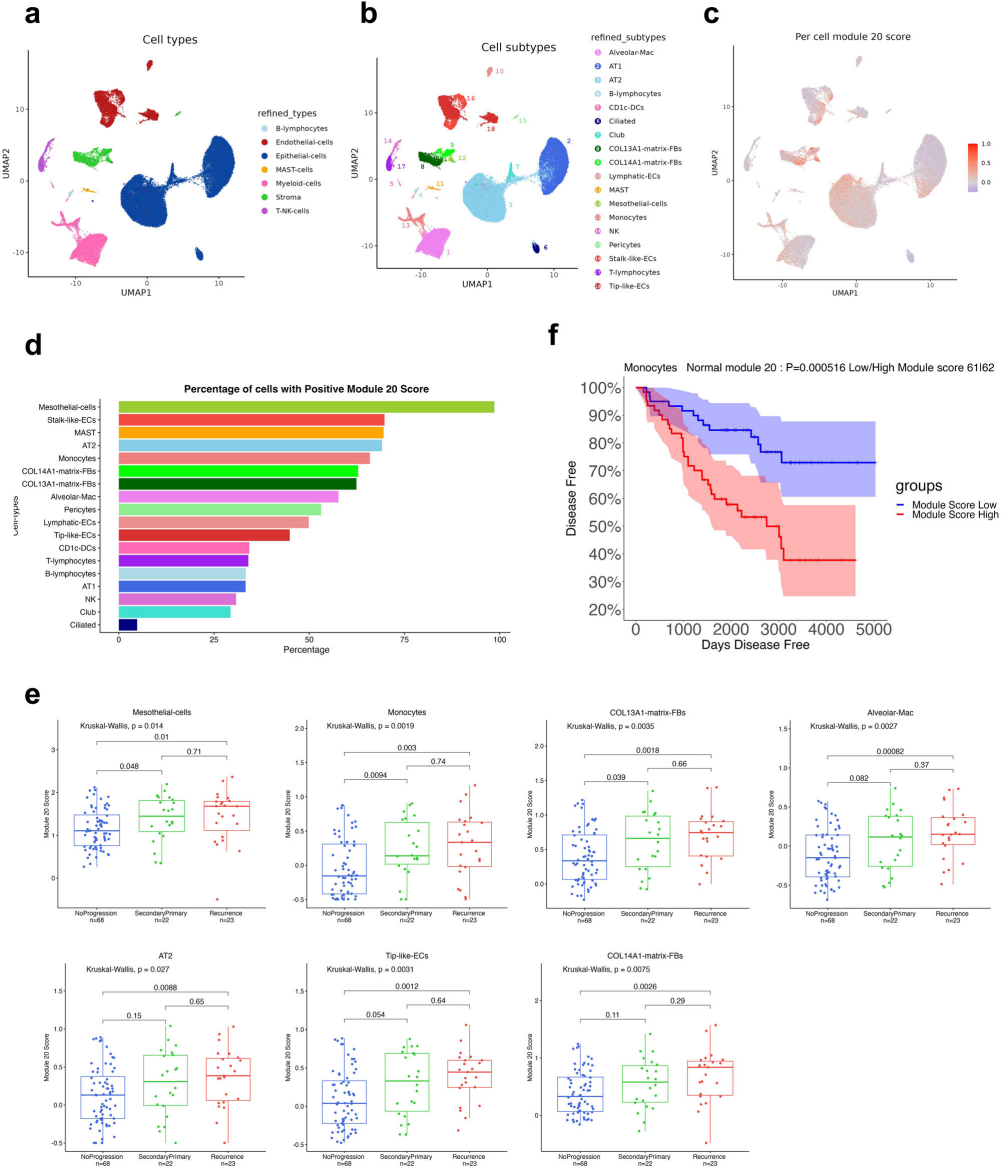
Single-nucleus RNA-seq analysis of tumor-adjacent normal tissue. **a** UMAP visualization of all 51,416 adjacent normal nuclei, color-coded based on the broad cell type annotation. **b** UMAP visualization of all 51,416 adjacent normal nuclei, color-coded based on the cell subtype annotation. **c** UMAP colored by module 20 score (calculated per nucleus). **d** Percentage of cells with a positive module 20 score in each cell subtype. **e** Cell subtypes with significantly upregulated expression of the module 20 signature in patients that eventually progress; statistical significance is calculated using the Mann–Whitney *U* test (two-sided; the Holm method was used to adjust *p*-values). Boxplots show medians (horizontal line in each box), interquartile ranges (boxes), 1.5 interquartile (whiskers) and each point represents a patient. **f** Kaplan–Meier curve for disease-free survival using the monocytes expression profile to calculate module 20 high (*n* = 62) and low (*n* = 61) groups. 95% confidence interval was also shown in shaded blue and red. *P*-value determined by the log-rank test.

To identify the cell types contributing to the expression of module 20, we utilized single-nucleus RNA-sequencing (snRNA-seq) to analyze the TAN tissue of our early-stage lung adenocarcinoma-matched tumor-normal cohort. We profiled 23 tumor and 23 matched TAN samples (see Methods for details). Following post-sequencing quality control we were left with 18 tumor and 15 normal snRNA-seq samples (112,626 nuclei). Genotyping analysis of the snRNA-seq data confirmed that these samples match the patient samples used for bulk RNA-seq (Supplementary Fig. 9). Cells were annotated based on a previous study of lung adenocarcinomas which included normal lung as control^23^ (see Methods). Focusing on the TAN samples (51,416 nuclei) (Supplementary Fig. 10a), we identified all major cell types: epithelial cells, stromal cells, endothelial cells, myeloid cells, T-NK cells, B lymphocytes and MAST cells (Fig. 6a). The distinct cell lineages were further delineated into more granular subpopulations (Fig. 6b, Supplementary Fig. 10b). Epithelial cells were divided into four subtypes: alveolar type 1 and 2 cells (AT1/AT2), club cells and ciliated cells. Stromal cells were divided into four subtypes: mesothelial cells, COL13A1 and COL14A1 matrix fibroblasts (FBs), and pericytes. Endothelial cells (ECs) were divided into three subtypes: lymphatic, stalk-like and tip-like ECs. Myeloid cells were divided into three subtypes: alveolar macrophages, monocytes, and CD1c DCs. The tumor samples (61,210 nuclei) consisted of the same cell types, lacked mesothelial cells, and included tumor cells, which were identified based on a high CNV score calculated based on inferCNV^24^ analysis (see Methods).

### Module 20 is activated in multiple cell types in the tumor-adjacent normal of patients that progress

To test which cell types in the tumor-adjacent normal lung have elevated expression of genes in module 20, we calculated a module 20 score per cell (Fig. 6c). We observed that the cell type that expressed the highest levels of module 20 genes were mesothelial cells, followed by fibroblasts, monocytes, stalk-like ECs, MAST cells, and alveolar macrophages (Fig. 6d and Supplementary Fig. 10c). Recently, mesothelial cells have been shown to form antigen-presenting cancer-associated fibroblasts (apCAFs), which in turn induce naive CD4+ T cells into regulatory T cells in pancreatic cancer^25^. Activation of the module 20 gene signature in AT2 cells (and not in AT1 cells) is also interesting because AT2 cells have been shown to be the cell of origin for lung adenocarcinoma^26^. To further investigate the activation of the module 20 signature in the tumor-adjacent normal tissue of the entire patient cohort, we applied BayesPrism^27^, a Bayesian statistical model that uses single-cell reference to deconvolve bulk RNA-seq expression. Based on our snRNA-seq data, BayesPrism inferred the cell-type composition of our larger bulk RNA-sequencing cohort (Supplementary Fig. 10d). Overall, we found that the relative abundance of mesothelial cells and monocytes, and to a lesser extent of stalk-like ECs, correlated highly with the module 20 score calculated from the bulk RNA-seq data, suggesting an increased production of mesothelial cells in lung tissue with increased overall TNF-α and NFκΒ signaling (Supplementary Fig. 10e). We then tested which cell types have upregulated expression of the module 20 signature in patients that progress. For this analysis, we used the inferred gene expression for each cell type in each patient. The results show a concomitant increase, in multiple cell types, of the module 20 score in patients that eventually develop a second primary or recurrence (Fig. 6e). Notably, we performed the same analysis on the matched tumor samples (Supplementary Fig. 11a–d) and we did not observe any significant differences in module 20 score between tumor samples from patients who progressed and those who did not in any of the cell types (Supplementary Fig. 11e).

We investigated the prognostic relevance of specific cell types within the TAN expressing module 20. Using the deconvoluted bulk RNA-seq, we identified significant differences in module 20 scores between the progression and no progression groups. Notably, monocytes exhibited a significant difference in module 20 scores (*p*-value < 0.01) (Fig. 6f), suggesting their potential as a valuable prognostic indicator. These findings highlight the importance of considering individual cell types within the TAN expressing module 20 and support the notion that they may offer additional prognostic power beyond the overall bulk module 20 score.

## Discussion

Early-stage lung adenocarcinoma is typically treated by surgical resection of the patient’s tumor. While in the majority of cases early intervention can lead to cure, approximately 30% of patients present with disease progression and eventually most of them eventually succumb to metastatic disease. Despite intense efforts to map the genetic landscape of early-stage lung tumors, there has been limited success in discovering accurate biomarkers that can predict progression-free survival. To address this significant unmet need, we proposed that the tumor-adjacent lung of early-stage lung adenocarcinoma patients is an unexplored source of potential biomarkers, and used a unique matched tumor and tumor-adjacent lung adenocarcinoma treatment naive patient cohort to identify molecular signatures associated with progression. To our knowledge, this is the largest such cohort both by size (number of patients) and follow-up time. We profiled both tumor and matched tumor-adjacent specimens using DNA and RNA sequencing and showed that gene expression in tumor-adjacent tissue is the best predictor of disease progression. Tumor heterogeneity across patients is a plausible explanation for this observation: although there are certainly frequently mutated driver genes in lung adenocarcinoma, such as *EGFR*, *KRAS* or *STK11*, only a minority of patients’ tumor is found positive for each of these mutations. Furthermore, co-occurring mutations and the presence of multiple tumor clones and subclones further complicate the already complex mutational landscape. Consequently, there is also a large diversity of transcriptional programs and pathways that are deregulated in tumors. This variability in the tumor transcriptomes is typically supported by PCA plots of tumor and normal samples: tumors are more scattered while normal samples cluster closer together. Therefore, because of the lack of commonly deregulated pathways across tumors, it is not surprising that models of disease progression that are based on tumor only are not accurate. By contrast, we found that tumor-adjacent tissue has a less diverse transcriptional profile, independent of the underlying driver mutations found in the adjacent tumor. This observation leads to the hypothesis that a common set of pathways may be activated in the tumor-adjacent tissue of patients that are at high risk for progression. Indeed, unsupervised discovery of co-expressed gene modules using bulk RNA-sequencing data obtained from our matched cohort uncovered an inflammatory signature (module 20) that can stratify patients into high and low risk groups independent of the underlying mutations found in their tumors. Intriguingly, we demonstrated that the module 20 signature is also associated with poor outcome in several other cancer types, suggesting that a common set of pathways is activated in the tumor-adjacent tissue of tumors that eventually progress. Further supporting our hypothesis, previous studies in other cancers have also suggested that tumor-adjacent tissue has distinct features that could provide prognostic information: hippo-related gene expression in hepatocellular carcinoma^28^, elevated mRNA levels of thymidylate synthase, vascular endothelial growth factor, and *EGFR* in rectal cancer^29^, different genes being expressed in prostate cancer^30^, and suppression of DMBT1 by cancer cells in squamous cell carcinomas^31^. Another study found that pathways shared among normal tissue adjacent to tumor are altered across different tumor types and suggests that pro-inflammatory signals from the tumor leads to the stimulation of an inflammatory response in the adjacent endothelium^32^. Moreover, specifically in lung cancer, the concept of “field cancerization” has been explored by Spira et al. with their investigations which demonstrate the utility of transcriptomic profiles from proximal airways as an adjunct to routine bronchoscopy for the diagnosis of the indeterminate pulmonary nodule^20^. In line with our results, there is an increased appreciation of the immune microenvironment in the treatment of resectable non-small cell lung cancers, driven by the progression-free survival benefit of neoadjuvant chemo-immunotherapy^33^. Taken together, our findings suggest that the proposed inflammatory signature may be used as a potential indicator of future recurrence events. However, given that the sensitivity and specificity reported here are relatively modest, larger studies will be necessary to further validate and improve upon these findings, and understand the functionality in light of new therapies specifically targeting the immune microenvironment.

Finally, analysis of the transcriptome of tumor-adjacent lung at single-cell resolution revealed that these inflammatory pathways are activated in specific cell types, mostly mesothelial cells, followed by stalk-like ECs, MAST cells, and alveolar type 2, and that characterization of the module 20 score in those specific cell types can further improve progression prediction. Two major pathways were identified as highly enriched in the module 20 signature: (1) the TNF-α pathway with genes IL6; JUNB, IRF1, SELE, and BCL3 overexpressed in patients who eventually progressed, and, (2) the IL17 pathway with genes JUND, TNFAIP3 and IL6 overexpressed. These two pathways suggest the provocative idea that patients with early-stage lung cancer may benefit from neoadjuvant therapy after their tumors are resected, such as TNF-α blockers or IL-17 inhibitors. Alternatively, the activation of inflammatory pathways in tumor-adjacent tissue may indicate that micrometastases have already occurred at undetectable levels. In such a scenario, there is evidence that blocking inflammation can help eradicate micrometastasis^34^. It is worth pointing out that the TAN may exhibit different molecular or immune features depending on the distance of the sampled TAN from the tumor. Certainly, a prospective study in which the distance in the collapsed resected tumor-associated normal lung is recorded at different areas, and then compared via digital spatial profiling to the tumor itself may give insight for TAN distance and prognosis, but this is beyond the scope of the current study. In conclusion, our studies suggest that molecular profiling of tumor-adjacent tissue can identify patients that are at high risk for progression and may help indicate appropriate neoadjuvant therapies for patients at risk.

## Methods

### Ethics statement

All patients were resected between 2006 and 2015 after signing informed consent for the New York Langone Health IRB continuously approved protocol i8896 C24 (The NYU Lung Cancer Biomarker Center approved May 6, 2020–April 17, 2024).

### Statistics and reproducibility

In our study, we ensured a balanced representation of participants, with an even distribution based on biological sex, incorporating equal numbers of male and female participants. In addition, our cohort spanned a wide age range, ensuring robust representation across various age groups. Upon analysis, we found consistent outcomes across all participants. Specifically, neither biological sex nor age exhibited any statistically significant influence on progression outcomes. These findings emphasize that, within the context of our study, other potential factors or variables may be more pivotal in determining progression than age or sex.

The participants in our study were all stage I lung adenocarcinoma patients from NYU Langone Health, each providing matched tumor and tumor-adjacent normal (TAN) tissue samples. The recruitment process did not present any discernible biases. It is noteworthy to mention that these participants had not undergone any cancer treatment prior to their surgeries, including radiation, immunotherapy, or chemotherapy. Participants’ race was determined based on self-identification. Furthermore, participants were not compensated.

Concerning sample size determination, no statistical methods were utilized beforehand to predetermine the sample size. We ensured transparency in our analyses, and no data was excluded from our results for reasons other than quality control. We used external datasets (TCGA) to demonstrate that the same score could stratify patients across cancer types. For the purpose of this study, patients were categorized based on their recurrence (either having no recurrence or recurrence) and progression status (secondary primary, locoregional, or systemic). There was no randomization or blinding involved in our study since a direct classification of each patient was a requisite.

### Specimen collection

Snap frozen Stage I lung cancer tumor and matching adjacent lung specimens (within the same lobe, segment, or wedge resection) from 143 patients having R0 resection with lymph node dissection were prospectively collected and archived at −80 ^o^C from 2005 to 2015 under an IRB approved NYULH protocol (i8896). Patients included in the study at no time prior to surgery ever received any treatment for cancer (i.e., radiation, immunotherapy, or chemotherapy). The resected tumors were from lobectomy wedge resections or segmentectomies and the matched TAN tissue was at least 3–4 cm from the edge of the tumor. Subjects were assessed postoperatively with an in-person clinic visit and surveillance chest CT every three months after surgery for two years, every six months for the third year and then yearly.

### Histologic characterization of tumors and TANs

Histological sections of the pulmonary adenocarcinomas were evaluated in formalin fixed paraffin embedded tissue. The percentage of each histological growth pattern (lepidic, acinar, papillary, solid, micropapillary, and complex glandular patterns (cribriform and fused glands) were recorded in 5% increment for each tumor to a sum of 100% as suggested by the current WHO classification of lung tumor^35^. The TAN samples were obtained from NYU patients whose tumors were resected by lobectomy wedge resections or segmentectomies. In a clinical setting, normal lung is routinely sectioned within 3 and more than 3 cm and submitted for pathological analysis. The samples used in this study followed this protocol. There was no evidence of tumor on the TAN slides, and as a result all these tumors were classified as stage 1. For downstream DNA-seq and RNA-seq analysis we used matching TAN samples that were at least 3–4 cm from the edge of the tumor.

### Determination of new primaries

The determination of second primaries typically follows several steps. We first apply the Martini-Melamed criteria^36^. Metachronus tumors are considered recurrent if they have similar morphology, are discovered within two years of original diagnosis, and the original tumor had a positive intervening lymph node or lymphovascular or pleural invasion. A tumor that does not fulfill these criteria is considered a new primary. In our lung adenocarcinoma study, the determination of morphologic similarity was made through a comprehensive subtyping and grading of the tumor. However, even a comprehensive analysis of morphology and subtype composition as well as thorough consideration of clinical information may sometimes fail to identify second primaries and further analysis using molecular profiling would certainly provide additional information in some cases.

### DNA sequencing

DNA sequencing of lung tumors, adjacent normal lung samples, and matched normal DNA extracted from blood, was performed using CLIA certified, clinically validated NYU Genome PACT assay for analysis of mutations and copy number changes. NYU Genome PACT is NYS approved, FDA-cleared custom-built, hybrid capture NGS assay analyzing all exons of 607 genes and TERT promoter, using IDT probes, sequenced on Illumina NextSeq 550 system (Illumina, San Diego, CA), with starting DNA input 200 ng, and average depth of sequencing 300x.

### DNA sequencing analysis

Sequencing results were demultiplexed and converted to FASTQ format using Illumina bcl2fastq (v2.0) software. The FASTQ files were processed using Seq-N-Slide (v22.01) pipeline^37^. The reads were adapter and quality trimmed with Trimmomatic (v0.39)^38^ and then aligned to the human reference genome (build hg38/GRCh38) using the Burrows-Wheeler Aligner with the BWA-MEM (v0.7) algorithm^39^. Low confidence mappings (mapping quality <10) and duplicate reads were removed using Sambamba (v1.0)^40^. Further local indel realignment and base-quality score recalibration were performed using the Genome Analysis Toolkit (GATK) (v3.8)^41^. Somatic variants in matched samples were called with Mutect (v4.1.9)^42^ and Strelka (v2.9.10)^43^. ANNOVAR (v2017Jul16)^44^ was used to annotate variants with genomic context such as functional consequence on genes and identify presence in public variant databases. The mean depth of coverage across all samples was 935X. Variant calls required >1% VAF, a minimum of 100 total reads, 5 alt reads, and a VAF >5 times that of a matched normal blood. To further reduce the likelihood of false positives, only known somatic variants present in Catalogue Of Somatic Mutations in Cancer (COSMIC)^45^ (v94) and with a population frequency of <0.1% based on gnomAD^46^ (v2.1.1) were retained.

### RNA sequencing

The quantity and quality of total RNA was assessed on a 2100 BioAnalyzer instrument (Agilent Technologies, Inc.). 1 ng of total RNA was used to prepare libraries using Trio RNA-Seq library prep kit (Tecan Genomics, Inc., part number 0506-96, mammalian rRNA Deplete) following the manufacturer’s instructions. Briefly, the library prep consists of the following steps: DNase treatment to remove genomic DNA, first strand and second strand cDNA synthesis from the input RNA, single primer isothermal amplification (SPIA) of the resultant cDNAs, enzymatic fragmentation and construction of unique barcoded libraries, PCR library amplification and a final step to remove rRNA transcripts. The Agencourt AMPure XP bead (Beckman Coulter) purified libraries were quantified using qPCR and the size distribution was checked using Agilent TapeStation 2200. The libraries were pooled and run on an Illumina S4 flow cell on a NovaSeq as paired end 100.

### RNA sequencing analysis

Sequencing results were demultiplexed and converted to FASTQ format using Illumina bcl2fastq (v2.0) software. The FASTQ files were processed using Seq-N-Slide (v22.01) pipeline^37^. The sequencing reads were adapter and quality trimmed with Trimmomatic (v0.39)^38^ and then aligned to the human reference genome (build hg38/GRCh38) using the splice-aware STAR aligner (v2.7.3)^47^. The featureCounts (v1.6.3) program^48^ was utilized to generate counts for each gene based on how many aligned reads overlap its exons. Useable samples were defined as those with more than 30% uniquely mapped reads, <50% of bases aligned to rRNA sequences, and more than 5 million assigned counts. The counts were then normalized and used to test for differential expression using negative binomial generalized linear models implemented by the DESeq2 (v1.40.2) R package^49^.

### Clustering patient samples by genotype

Sample relatedness to ensure that data from the same patients was correctly labeled was computed using Somalier (v0.2.18)^50^, which analyzes ancestry based on common variants across all human populations and calculates pairwise coefficient of relationship. Sample pairs with <50 sites were excluded. Pairwise relatedness values <0 were set to 0 for visualization and hierarchical clustering.

### Machine learning classifier for 5-year recurrence

We trained a logistic regression model with elastic net penalty to classify patients that recur vs those that do not based on their gene expression. Our machine learning method combines hard filtering (200 most variable genes) with soft filtering (elastic net regression) and therefore we utilized a nested cross-validation scheme to get an unbiased estimate of its performance and avoid data leakage. At a high-level we use the following steps:

1. For a given outer train-test 10-fold split:
  - we selected top-N genes based on the training data (details below)
  - we used an inner 10-fold on the training data to optimize the parameters (details below)
  - the optimal model, as determined by inner cross-validation, was then applied to the test data of that split
2. Then, we combined the predictions of the individual test sets across all splits (thus covering the entire cohort), and we used these predictions to:
  - generate ROC curves and calculate AUCs
  - split patients into high-low risk based on the recurrence prediction and generate Kaplan–Meier plots.

More specifically, the outer CV-split defines the X matrix while the inner CV fits the β, λ, α parameters. In more detail, in the outer loop we identify the 200 most variable (according to the median absolute deviation) genes of the training split and use them to fit a logistic regression model elastic net regularization. The fitting of the model takes place in the inner (10-fold) cross-validation using 21 potential values for *α* (a_n = (*n*/20)^2^ for *n* = 0, …, 20) while the *λ* values were automatically adjusted by the glmnet (v4.1) package. The best model, in terms of mean cross-validated error, from the inner CV is then used to classify the test cases of the outer split. Finally, the predicted probability of recurrence for all the test sets were combined and used to estimate the ROC curves and plot the Kaplan–Meier curves. The loss function for the logistic regression with elastic net penalty is shown below:

1 $${\min }_{{\beta }_{0},\beta }\frac{1}{N}\mathop{\sum}\limits_{i}\log L\left({y}_{i},{\beta }_{0}+{\beta x}_{i}\right)+\lambda \left[\left(1-\alpha \right)\frac{\|\beta \|^{2}_{2}}{2}+\alpha {\|\beta \|}_{1}\right]$$

Where: ${{{{{\rm{L}}}}}}(y,\hat{y})$ is the likelihood of the binomial distribution and *β*_0_,*β* are the parameters to be tuned, *x*_*i*_ is the expression vector for patient i whose dimensions are the 200 most variable genes across all patients, *λ* controls the regularization penalty, *α* the trade-off between lasso and ridge regression.

### Gene co-expression analysis

The expression counts were transformed using variance stabilizing transformation (VST) implemented in DESeq2. Only protein-coding genes as identified by GENCODE were retained. Genes were further subset to the 10,000 most variable. Principal components analysis (PCA) was performed on a data matrix of values that were scaled and centered for each gene. The first 10 PCs were used for clustering and UMAP visualization. Gene modules were determined using Partitioning Around Medoids (PAM) clustering implemented in cluster (v2.1.4) R package with a *k* = 20 and pamonce = 5. UMAP was generated using the uwot (v0.1.16) R package with *n*_neighbors = 10 and min_dist = 0.3. The module scores were defined as the average of the z-scores of genes within each module.

### Association of module scores with demographic, clinical, histologic, genetic and outcome variables

For the 20 module scores for all patients, we computed the correlation of them with different demographic, histological, clinical and mutational status features. Then the correlation significance level of each module vs. other features was plotted as a dot plot using R package ggplot2 (v3.3.6).

### Nucleus isolation and sequencing

Nuclei were prepared for 10x Genomics-based single nuclei RNA seq analysis according to a previously published protocol^51^. Briefly, each frozen sample was thawed and macerated in CST buffer for 10 min, filtered (70 micron pluriStrainer) and spun at 500 g for 5 min at 4 ^o^C to pellet nuclei. Nuclei were resuspended in the same buffer without detergent, filtered (10 micron pluriStrainer) and counted using AOPI on a Nexcelom Cellometer. Approximately 10,000 nuclei were loaded immediately into each channel of a 10x Chromium chip (10x Genomics) using 5-prime v1.1 chemistry according to the manufacturer’s protocol (10x Genomics #CG000208). The resulting cDNA and indexed libraries were checked for quality on an Agilent 4200 TapeStation and then quantified and pooled for sequencing on an Illumina NovaSeq 6000.

### snRNA-seq data preprocessing

Sequencing reads were trimmed of adapter sequences using cutadapt (v4.2)^52^. Barcode processing and gene quantification was performed with STARsolo (v2.7.3)^53^ using the GRCh38 human reference transcriptome (refdata-cellranger-GRCh38-3.0.0 provided by 10x Genomics). STARsolo pre-mRNA counts were used to generate the gene-barcode matrix. Further analysis including the identification of highly variable genes, dimensionality reduction, standard unsupervised clustering algorithms, and the discovery of differentially expressed genes was performed using Seurat (v4.0)^54^ and streamlined as an R package (available at https://github.com/igordot/scooter).

Nuclei were filtered to only include those with >500 detectable genes, >1000 UMIs, and <10% of transcripts coming from mitochondrial genes. The UMI counts were normalized by the total number of UMIs per nucleus, multiplied by a scale factor of 10,000, and log-transformed. Likely doublets/multiplets were identified and removed using the scDblFinder (v1.6.0) package^55^.

### Dimensionality reduction and annotation

To visualize the data, the dimensionality of the scaled integrated data matrix was further reduced to project the nuclei in two-dimensional space using PCA followed by uniform manifold approximation and projection (UMAP)^56^ using top 30 PCs and 30 nearest neighbors to define the local neighborhood size with a minimum distance of 0.3. The resulting PCs were also used as a basis for partitioning the dataset into clusters using a smart local moving (SLM) community detection algorithm^57^. A range of resolutions (0.1–10) was utilized to establish a sufficient number of clusters.

Nuclei were annotated using a previous study of lung adenocarcinomas as a reference^23^. Brain metastasis samples were removed from the reference dataset. SingleR (v1.6.1)^58^ annotation was performed on the aggregated cluster profiles using 86 clusters (resolution of 3) with cell type and cell subtype labels. The tumor cells were identified based on a high copy number variant (CNV) score determined by InferCNV (v1.11.2)^24^. This score was computed as the sum of all proportion_cnv columns (representing both gains and losses) in the output of the add_to_seurat inferCNV function. For each cell type, a threshold was established as the maximum CNV value observed in the TAN. Any cell exhibiting a CNV higher than the established threshold was classified as a tumor cell.

Further analysis was performed on 15 normal samples (51,416 nuclei) and 18 tumor samples (61,210 nuclei). To account for biological and technical batch differences between individual patients and scRNA-seq libraries, the Harmony (v0.0.1)^59^ integration method for merging datasets that identify pairwise correspondences between cell pairs across datasets to transform them into a shared space was utilized. To maintain the distinct transcriptional profiles of the tumor cells within the UMAP visualization, we applied Harmony batch correction exclusively to the non-tumor cells within the tumor samples, consistent with the approach delineated in previous publications^60–62^. Our cell annotation structure was further refined by defining the cell subtypes based on the majority cell subtype of each cluster. In addition, for the tumor cells, we adopted an overclustering approach for each cell type to ensure a finer resolution. After this overclustering, we employed a methodology analogous to what we utilized for TAN: we identified the majority cell subtype within each cluster and used that designation for the entire cluster. This approach ensures that our UMAP visualizations are both precise and representative, drawing from the inherent transcriptional landscape of the tumor cells while also leveraging refined clustering techniques. Seurat’s AddModuleScore function was used to quantify gene set expression in each nucleus.

### Bulk RNA-seq deconvolution

BayesPrism (v2.0) was used for deconvolution of the TAN and tumor bulk RNA samples^27^. Mitochondrial and ribosomal protein coding genes were excluded from deconvolution analysis. To increase the signal-to-noise ratio, we also removed lowly transcribed genes, leaving us with 19,816 genes. To reduce batch effects and decrease computational time, we retained only protein coding genes for a total gene count of 13,972 for the single-nucleus reference. Next, cells were labeled according to the cell subtypes as identified by a previous group^23^. For BayesPrism TAN analysis, the parameter key was set to NULL to indicate there were no malignant cells in the reference and all 21 cell types are treated equally. Final Gibbs theta values were used to estimate the fraction of each cell type. We extracted the posterior mean of each cell-type specific gene expression for the outputted count matrix, *Z* for every cell type. Next, we computed the z-score for all genes across our cell types of interest. The module 20 score was then defined as the average of z-scores of the module genes. A Mann–Whitney *U* test was run between the progression (second primary or recurrence) and no progression groups for each cell-type. *P*-values < 0.01 were considered statistically significant.

### Reporting summary

Further information on research design is available in the Nature Portfolio Reporting Summary linked to this article.

### Supplementary information

Supplementary Information

Reporting Summary

Description of Additional Supplementary Files

Dataset 1

Dataset 2

Dataset 3

Dataset 4

Dataset 5

Dataset 6

### Source data

Source Data

## Data Availability

All data generated and supporting the paper are available within this paper. The snRNA-seq and bulk RNA-seq were submitted to Gene Expression Omnibus (GEO) repository and can be accessed under GEO accession no. GSE229706. The processed DNA-sequencing, RNA sequencing, and single-nucleus data can be found on FigShare. Source data are provided with this paper.

## Code availability

The code for this paper is available at https://github.com/ninashenker/LUAD^63^.
