# Supplementary material for: Inflammation in the tumor-adjacent lung as a predictor of clinical outcome in lung adenocarcinoma
Source: Nat Commun. 2023 Nov 8;14:6764. doi: 10.1038/s41467-023-42327-x (PMC10632519; doi:10.1038/s41467-023-42327-x)
Supplement: Supplementary file 1 — Supplementary Information [file 41467_2023_42327_MOESM1_ESM.pdf]

Supplementary Information

Inflammation in the tumor-adjacent lung as a predictor of clinical outcome in lung adenocarcinoma

Igor Dolgalev[1,2,3,*], Hua Zhou[1,2,*], Nina Murrell[1,2,3,*], Hortense Le[1,3], Theodore Sakellaropoulos[1], Nicolas Coudray[2,3,4], Kelsey Zhu[1], Varshini Vasudevaraja[1], Anna Yeaton[5], Chandra Goparaju[6], Yonghua Li[7], Imran Sulaiman[7], Jun-Chieh J. Tsay[7], Peter Meyn[8], Hussein Mohamed[1], Iris Sydney[9], Tomoe Shiomi[9], Sitharam Ramaswami[1,8], Navneet Narula[1], Ruth Kulicke[10], Fred P. Davis[10], Nicolas Stransky[10], Gromoslaw A. Smolen[10], Wei-Yi Cheng[11], James Cai[11], Salman Punekar[12], Vamsidhar Velcheti[12], Daniel H Sterman[7,12], J.T. Poirier[12], Ben Neel[12], Kwok-Kin Wong[12], Luis Chiriboga[1], Adriana Heguy[1,8,12], Thales Papagiannakopoulos[1,12], Bettina Nadorp[1,2,3], Matija Snuderl[1,12], Leopoldo N. Segal[7,12], Andre L. Moreira[1,12], Harvey I. Pass[6,12,#], Aristotelis Tsirigos[1,2,3,12,#]

[1] Department of Pathology, NYU Grossman School of Medicine, New York, USA
[2] Applied Bioinformatics Laboratories, NYU Grossman School of Medicine, New York, USA
[3] Division of Precision Medicine, Department of Medicine, NYU Grossman School of Medicine, New York, USA
[4] Department of Cell Biology, NYU Grossman School of Medicine, New York, USA
[5] The Optical Profiling Platform at The Broad Institute of MIT And Harvard
[6] Department of Cardiothoracic Surgery, NYU Grossman School of Medicine, New York, USA
[7] Division of Pulmonary, Critical Care and Sleep Medicine, NYU Grossman School of Medicine, New York, USA
[8] Genome Technology Center, Office of Science and Research, NYU Grossman School of Medicine, New York, USA
[9] Center for Biospecimen Research and Development, NYU Grossman School of Medicine, New York, USA
[10] Celsius Therapeutics, Cambridge, Massachusetts, USA
[11] Pharma Research & Early Development Informatics, Roche Innovation Center New York, New Jersey, USA
[12] Laura and Isaac Perlmutter Cancer Center, New York University Langone Health, New York, New York

* These authors contributed equally

# Corresponding authors:
Aristotelis Tsirigos, Aristotelis.Tsirigos@nyulangone.org
Harvey I. Pass, Harvey.Pass@nyulangone.org

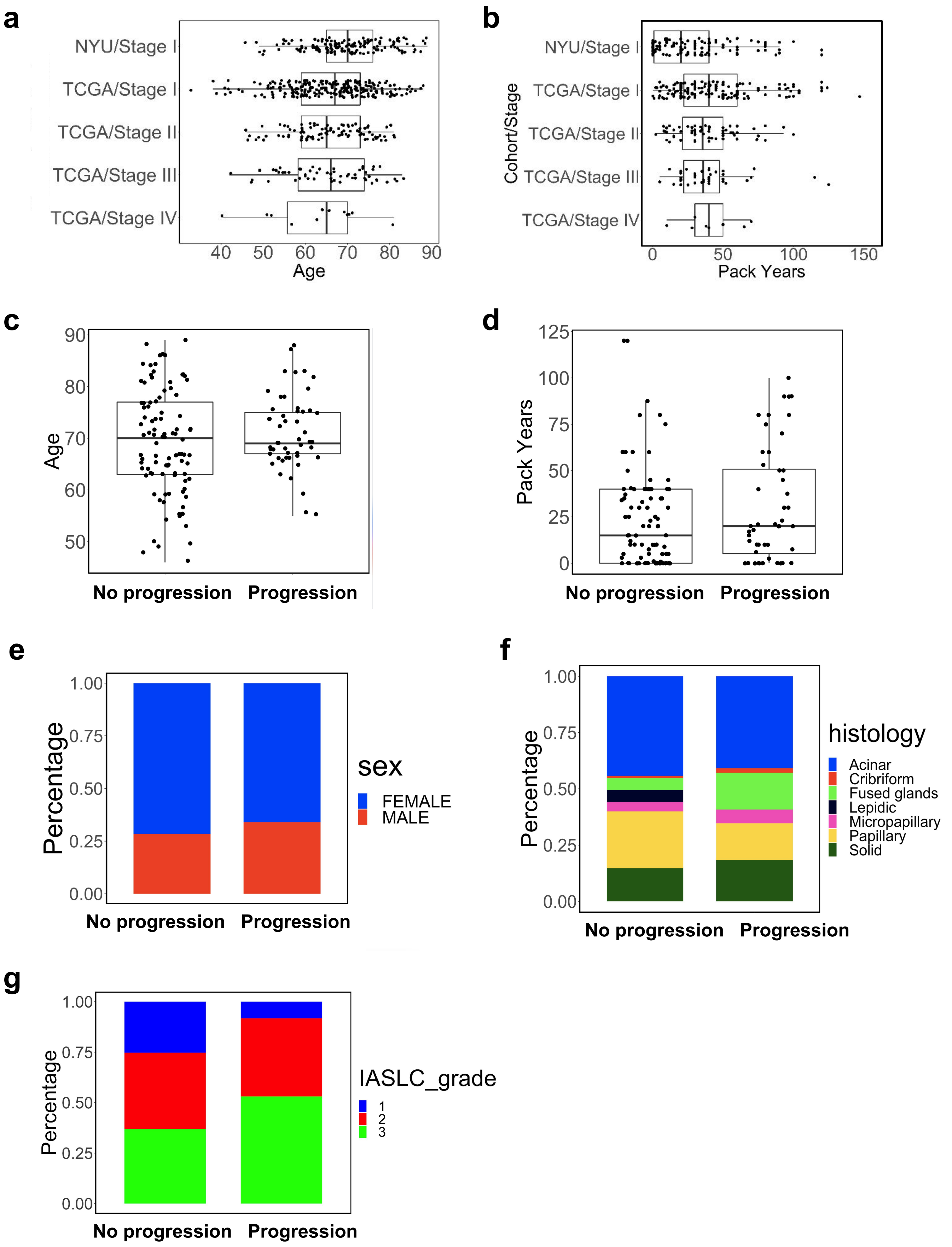
a
NYU/Stage I
TCGA/Stage I
TCGA/Stage II
TCGA/Stage III
TCGA/Stage IV
40 50 60 70 80 90
Age
b
Cohort/Stage
NYU/Stage I
TCGA/Stage I
TCGA/Stage II
TCGA/Stage III
TCGA/Stage IV
0 50 100 150
Pack Years
c
Age
90 80 70 60 50
No progression
Progression
d
Pack Years
125 100 75 50 25 0
No progression
Progression
e
Percentage
1.00 0.75 0.50 0.25 0.00
sex
FEMALE
MALE
No progression
Progression
f
Percentage
1.00 0.75 0.50 0.25 0.00
histology
Acinar
Cribriform
Fused glands
Lepidic
Micropapillary
Papillary
Solid
No progression
Progression
g
Percentage
1.00 0.75 0.50 0.25 0.00
IASLC_grade
1
2
3
No progression
Progression

Supplementary Figure 1. Additional cohort characteristics.

(a) Patient age distributions represented as boxplots in NYU (n=110) and TCGA cohorts (by stage, Stage I: n=243, Stage II: n=94, Stage III: n=61, Stage IV: n=12). Boxplots show medians (horizontal line in each box), interquartile ranges (boxes), 1.5 interquartile (whiskers) and each point represents a patient.

(b) Patient pack year distributions represented as boxplots in NYU (n=110) and TCGA cohorts (by stage, Stage I: n=243, Stage II: n=94, Stage III: n=61, Stage IV: n=12). Boxplots show medians (horizontal line in each box), interquartile ranges (boxes), 1.5 interquartile (whiskers) and each point represents a patient.

(c) Patient age by progression status represented as boxplots in NYU (n=145). Median, first and third quartiles are shown in boxplots,

(d) Patient pack year distributions by progression status represented as boxplots (NYU cohort only, n=145). Boxplots show medians (horizontal line in each box), interquartile ranges (boxes), 1.5 interquartile (whiskers) and each point represents a patient.

(e) Percentage of male and female patients by progression status (NYU cohort),

(f) Breakdown of histologic types by progression status (NYU cohort),

(g) Tumor grade by progression status (NYU cohort),

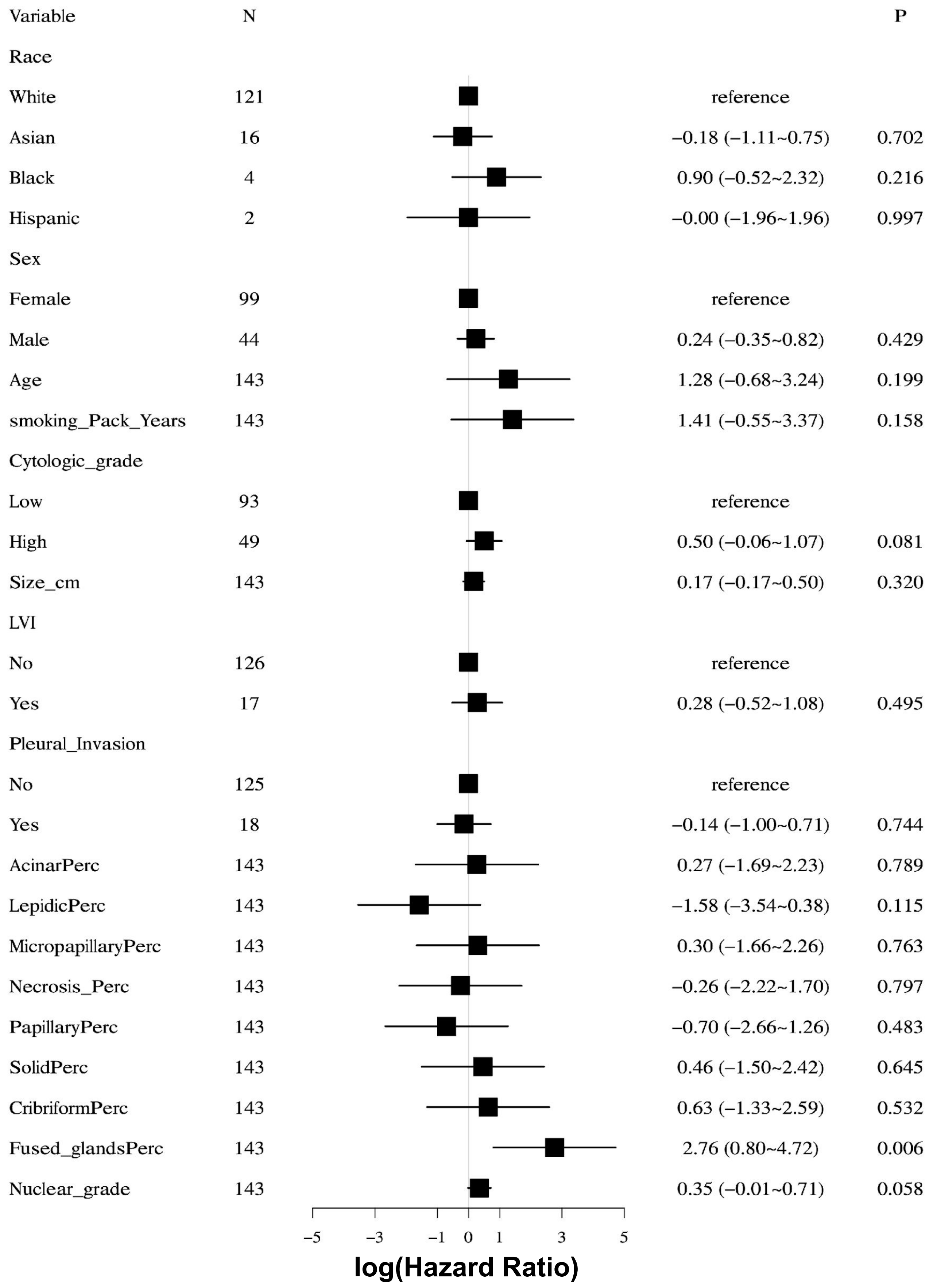

Variable N P
Race
White 121 reference
Asian 16 −0.18 (−1.11~0.75) 0.702
Black 4 0.90 (−0.52~2.32) 0.216
Hispanic 2 −0.00 (−1.96~1.96) 0.997
Sex
Female 99 reference
Male 44 0.24 (−0.35~0.82) 0.429
Age 143 1.28 (−0.68~3.24) 0.199
smoking_Pack_Years 143 1.41 (−0.55~3.37) 0.158
Cytologic_grade
Low 93 reference
High 49 0.50 (−0.06~1.07) 0.081
Size_cm 143 0.17 (−0.17~0.50) 0.320
LVI
No 126 reference
Yes 17 0.28 (−0.52~1.08) 0.495
Pleural_Invasion
No 125 reference
Yes 18 −0.14 (−1.00~0.71) 0.744
AcinarPerc 143 0.27 (−1.69~2.23) 0.789
LepidicPerc 143 −1.58 (−3.54~0.38) 0.115
MicropapillaryPerc 143 0.30 (−1.66~2.26) 0.763
Necrosis_Perc 143 −0.26 (−2.22~1.70) 0.797
PapillaryPerc 143 −0.70 (−2.66~1.26) 0.483
SolidPerc 143 0.46 (−1.50~2.42) 0.645
CribriformPerc 143 0.63 (−1.33~2.59) 0.532
Fused_glandsPerc 143 2.76 (0.80~4.72) 0.006
Nuclear_grade 143 0.35 (−0.01~0.71) 0.058
−5 −3 −1 0 1 3 5
log(Hazard Ratio)

Supplementary Figure 2. Cox regression on clinicodemographic variables.

Log of Hazard Ratio and 95% confidence interval were shown in forest plot.

a

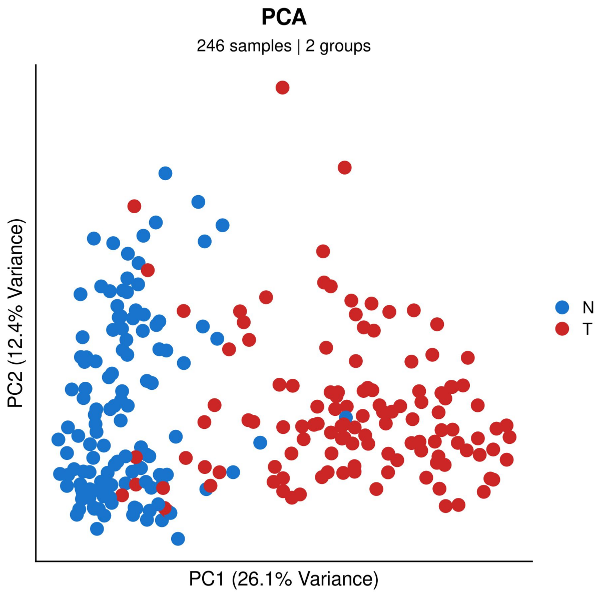
PCA
246 samples | 2 groups
PC2 (12.4% Variance)
PC1 (26.1% Variance)
N
T

b

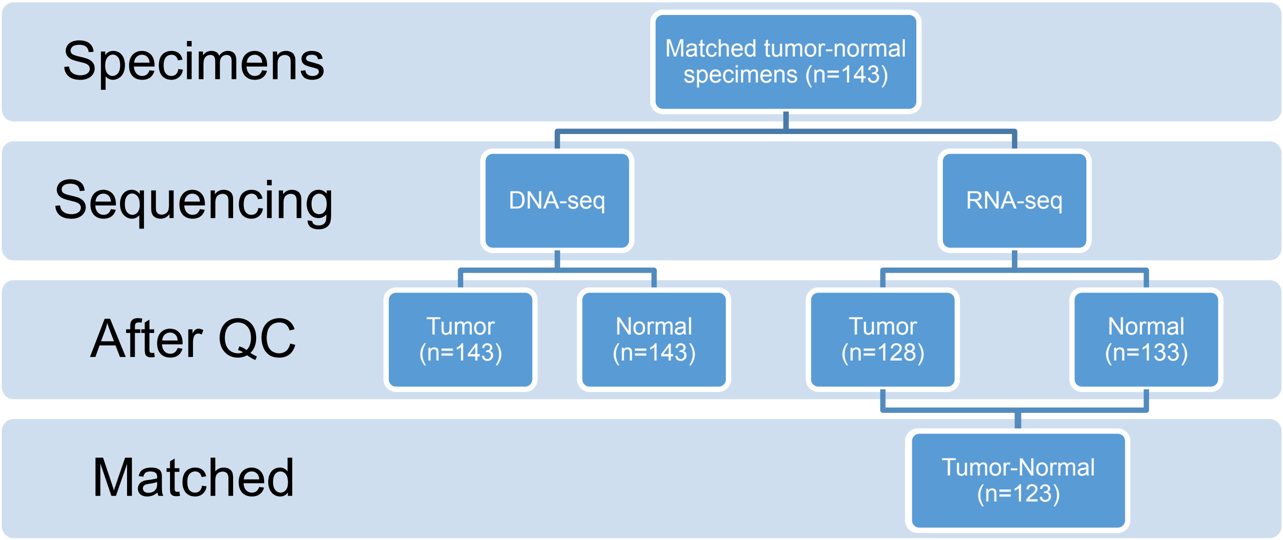
Specimens
Matched tumor-normal specimens (n=143)
Sequencing
DNA-seq
RNA-seq
After QC
Tumor (n=143)
Normal (n=143)
Tumor (n=128)
Normal (n=133)
Matched
Tumor-Normal (n=123)

Supplementary Figure 3. Sequencing and quality control workflow.

(a) PCA of tumor-normal RNA samples

(b) Number of normal and tumor samples with DNA-seq and RNA-seq

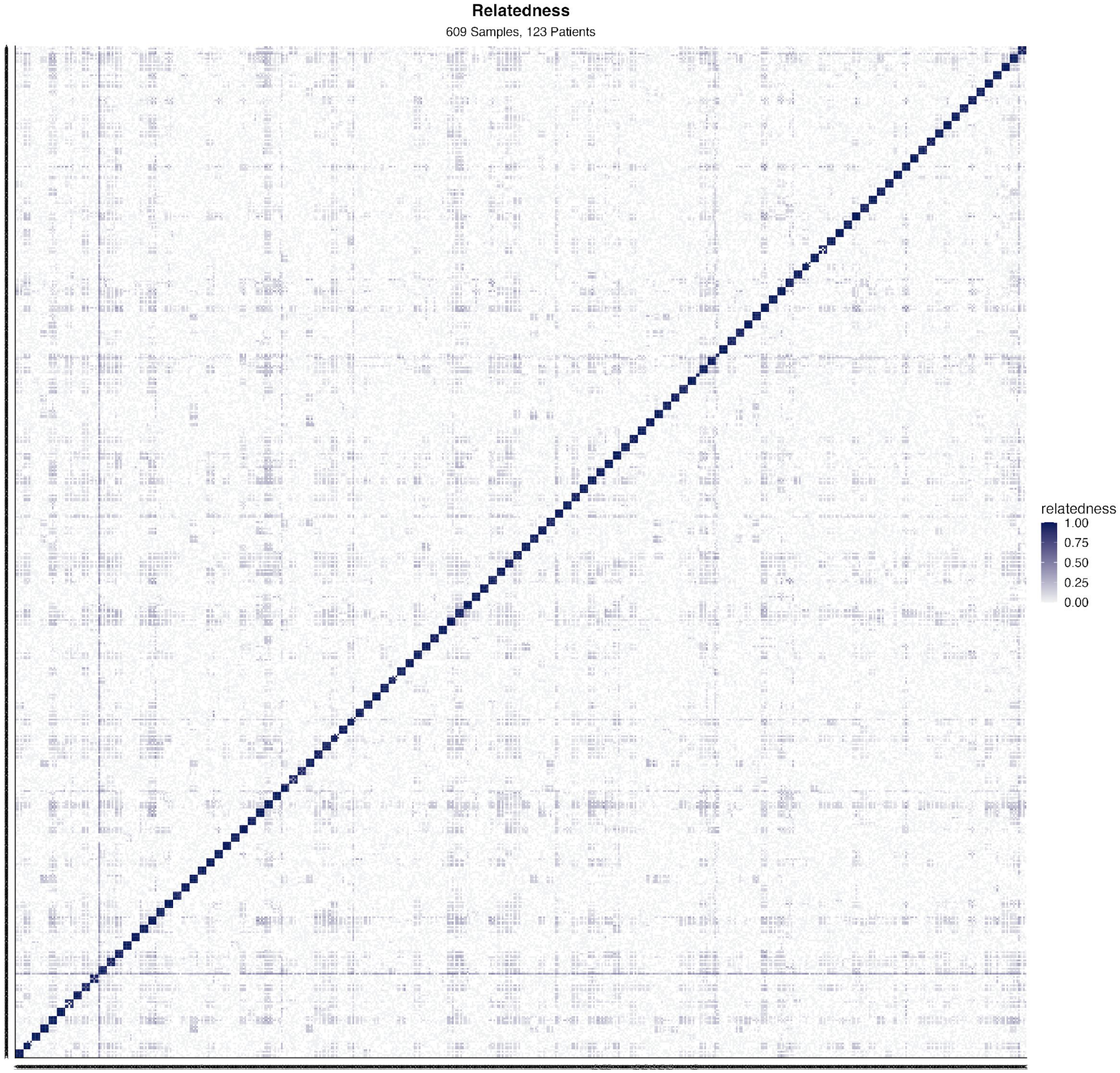
Relatedness
609 Samples, 123 Patients
relatedness
1.00
0.75
0.50
0.25
0.00

Supplementary Figure 4. Clustering of all patient samples by genotype. Pairwise similarity of variants called on all available samples. Each patient has up to 5 sequenced samples: DNA-seq of tumor, normal and blood samples, and RNA-seq of tumor, normal samples. Samples from the same patient cluster together as expected.

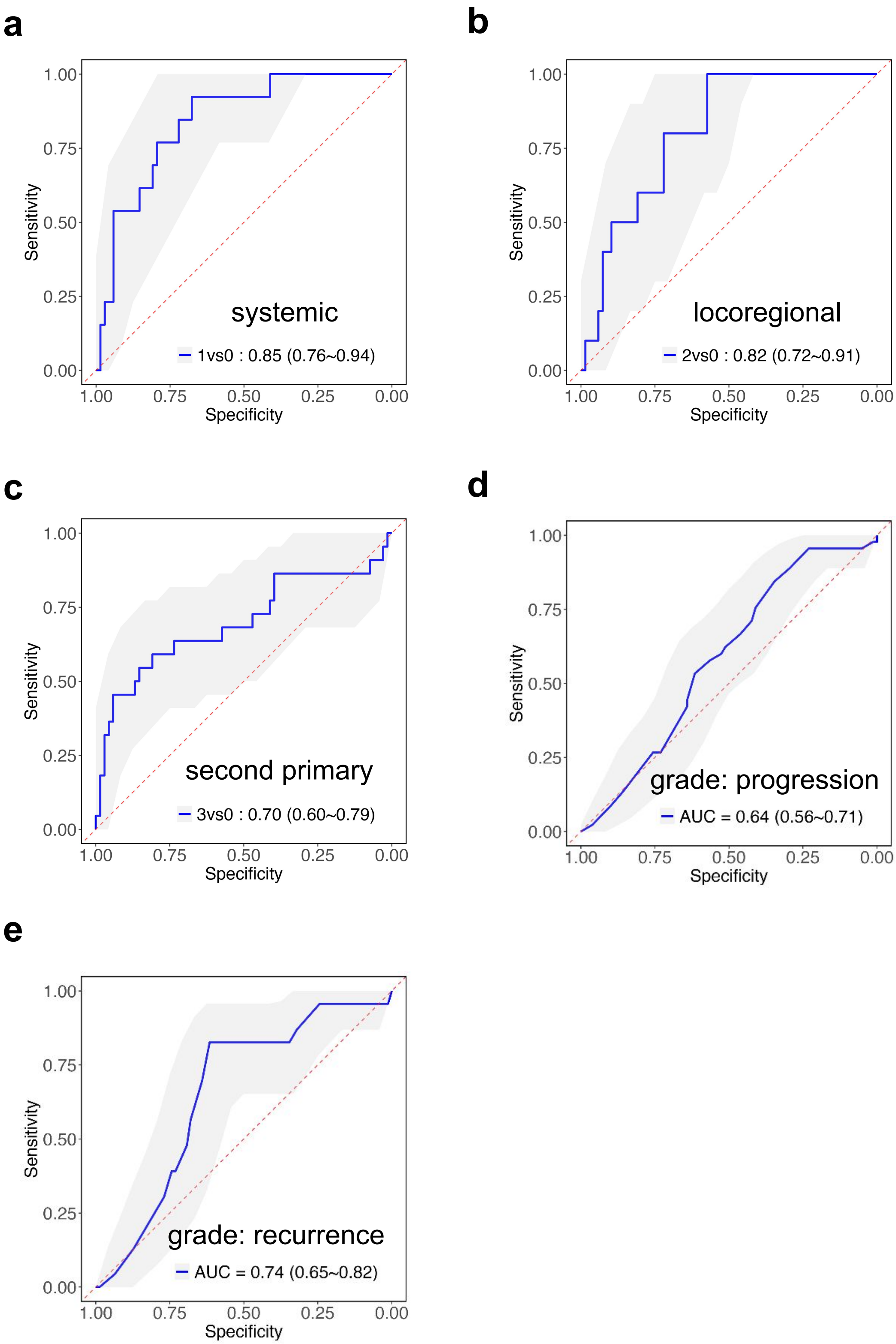
a
b
c
d
e
Sensitivity
Specificity
1.00
0.75
0.50
0.25
0.00
systemic
1vs0 : 0.85 (0.76~0.94)
locoregional
2vs0 : 0.82 (0.72~0.91)
second primary
3vs0 : 0.70 (0.60~0.79)
grade: progression
AUC = 0.64 (0.56~0.71)
grade: recurrence
AUC = 0.74 (0.65~0.82)

Supplementary Figure 5: Prognostic models based on TAN transcriptome and IASLC grade.

(a) Receiver-operating characteristic curve (ROC) for the prediction of LUAD systemic recurrence (1) vs no recurrence (0), 95% confidence interval was shown in gray.

(b) ROC curve for the prediction of locoregional recurrence (2), 95% confidence interval was shown in gray.

(c) ROC curve for the prediction of LUAD second primary tumor (3), 95% confidence interval was shown in gray.

(d) ROC curve for the prediction of LUAD progression based on IASLC grade, 95% confidence interval was shown in gray.

(e) ROC curve for the prediction of LUAD recurrence based on IASLC grade, 95% confidence interval was shown in gray.

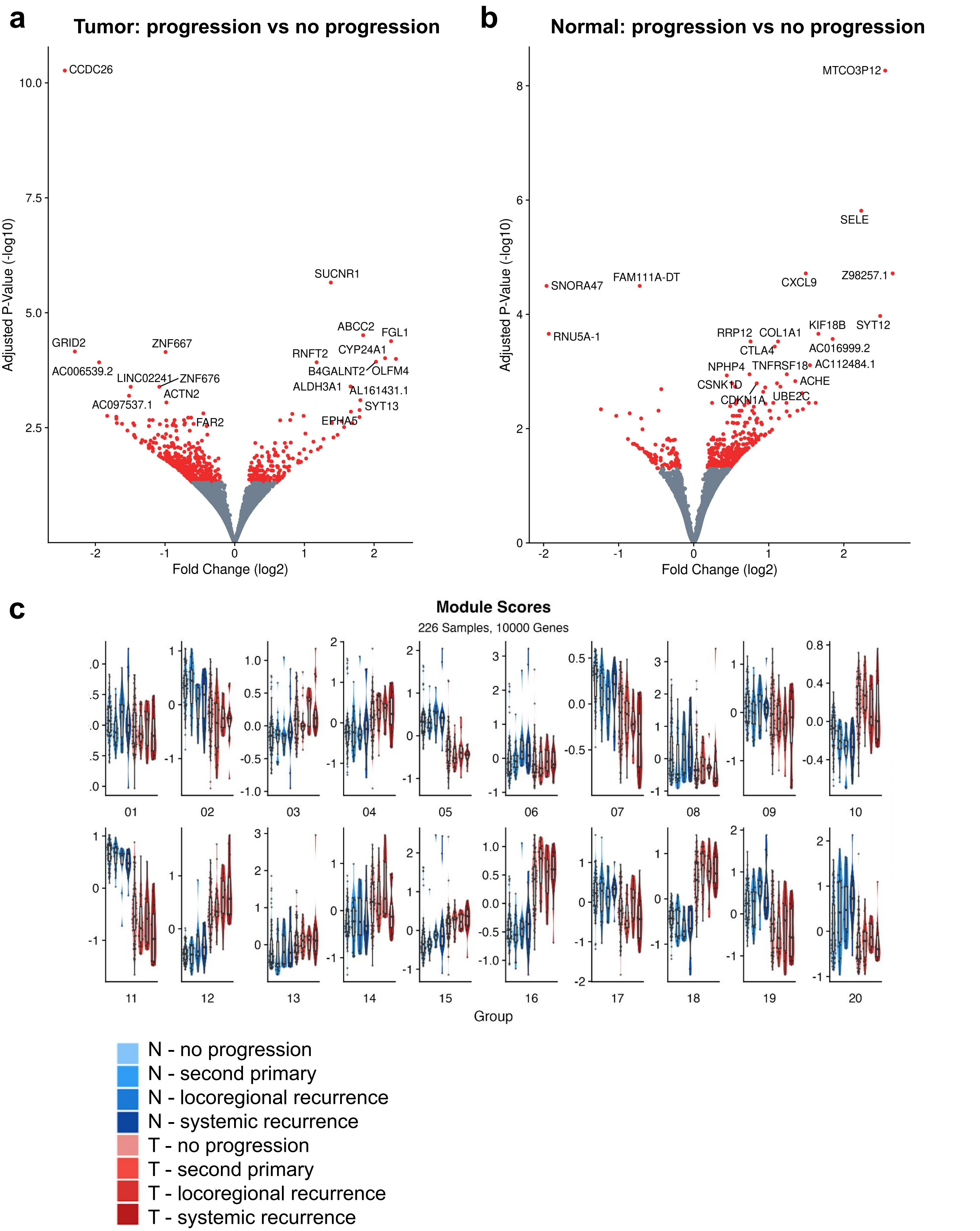
a
Tumor: progression vs no progression
Adjusted P-Value (-log10)
Fold Change (log2)
CCDC26
SUCNR1
ABCC2
FGL1
GRID2
ZNF667
RNFT2
CYP24A1
AC006539.2
LINC02241
ZNF676
B4GALNT2
OLFM4
ACTN2
ALDH3A1
AL161431.1
AC097537.1
SYT13
FAR2
EPHA5
b
Normal: progression vs no progression
Adjusted P-Value (-log10)
Fold Change (log2)
MTCO3P12
SELE
SNORA47
FAM111A-DT
CXCL9
Z98257.1
RNU5A-1
RRP12
COL1A1
KIF18B
SYT12
CTLA4
AC016999.2
NPHP4
TNFRSF18
AC112484.1
CSNK1D
ACHE
CDKN1A
UBE2C
c
Module Scores
226 Samples, 10000 Genes
Group
N - no progression
N - second primary
N - locoregional recurrence
N - systemic recurrence
T - no progression
T - second primary
T - locoregional recurrence
T - systemic recurrence

Supplementary Figure 6: Transcriptomic signatures of disease progression in stage I lung adenocarcinoma.

(a) Volcano plot representing the differential expression analysis comparing patients with progression vs no progression (tumor samples). The p-values are calculated with DESeq2 (Wald test with Benjamini and Hochberg multiple testing correction).

(b) Volcano plot representing the differential expression analysis comparing patients with progression vs no progression (normal lung samples). The p-values are calculated with DESeq2 (Wald test with Benjamini and Hochberg multiple testing correction).

(c) Boxplots comparing modules scores by progression type in tumor and TAN tissue in each module. Boxplots show medians (horizontal line in each box), interquartile ranges (boxes), 1.5 interquartile (whiskers) and each point represents a patient.

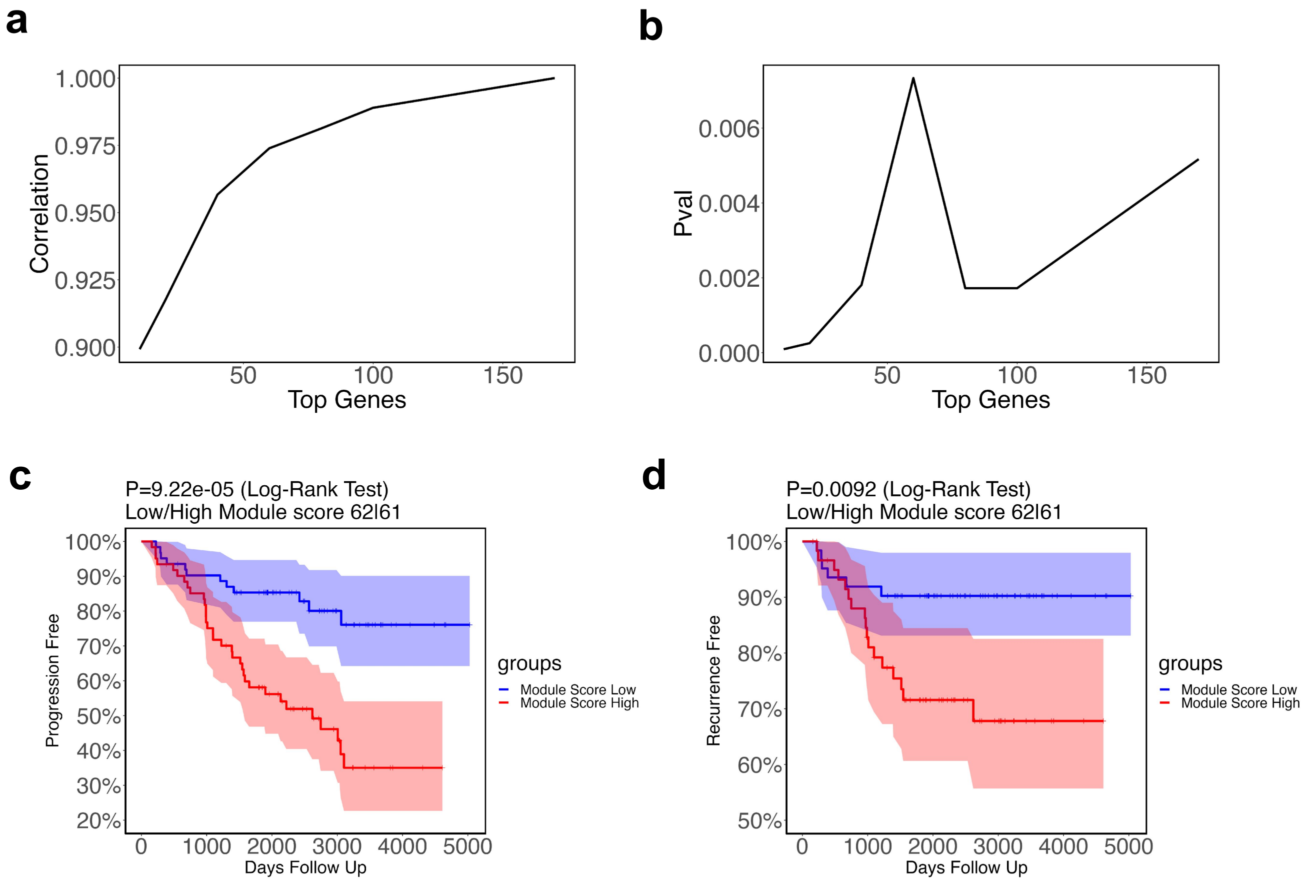
a
Correlation
1.000
0.975
0.950
0.925
0.900
50
100
150
Top Genes
b
Pval
0.006
0.004
0.002
0.000
50
100
150
Top Genes
c
P=9.22e-05 (Log-Rank Test)
Low/High Module score 62I61
Progression Free
100%
90%
80%
70%
60%
50%
40%
30%
20%
0
1000
2000
3000
4000
5000
Days Follow Up
groups
Module Score Low
Module Score High
d
P=0.0092 (Log-Rank Test)
Low/High Module score 62I61
Recurrence Free
100%
90%
80%
70%
60%
50%
0
1000
2000
3000
4000
5000
Days Follow Up
groups
Module Score Low
Module Score High

Supplementary Figure 7: Analysis of reduced module 20 signature.

(a) Correlation of top 10, 20, 40, 60, 80, 100, 170 module 20 genes with full module 20 signature.

(b) P-values for the correlations in panel (a).

(c) Kaplan-Meier progression-free survival curves of patients with high and low module 20 scores based only on the top 10 genes, 95% confidence interval was shown in shaded blue and red.

(d) Kaplan-Meier recurrence-free survival curves of patients with high and low module 20 scores based only on the top 10 genes, 95% confidence interval was shown in shaded blue and red.

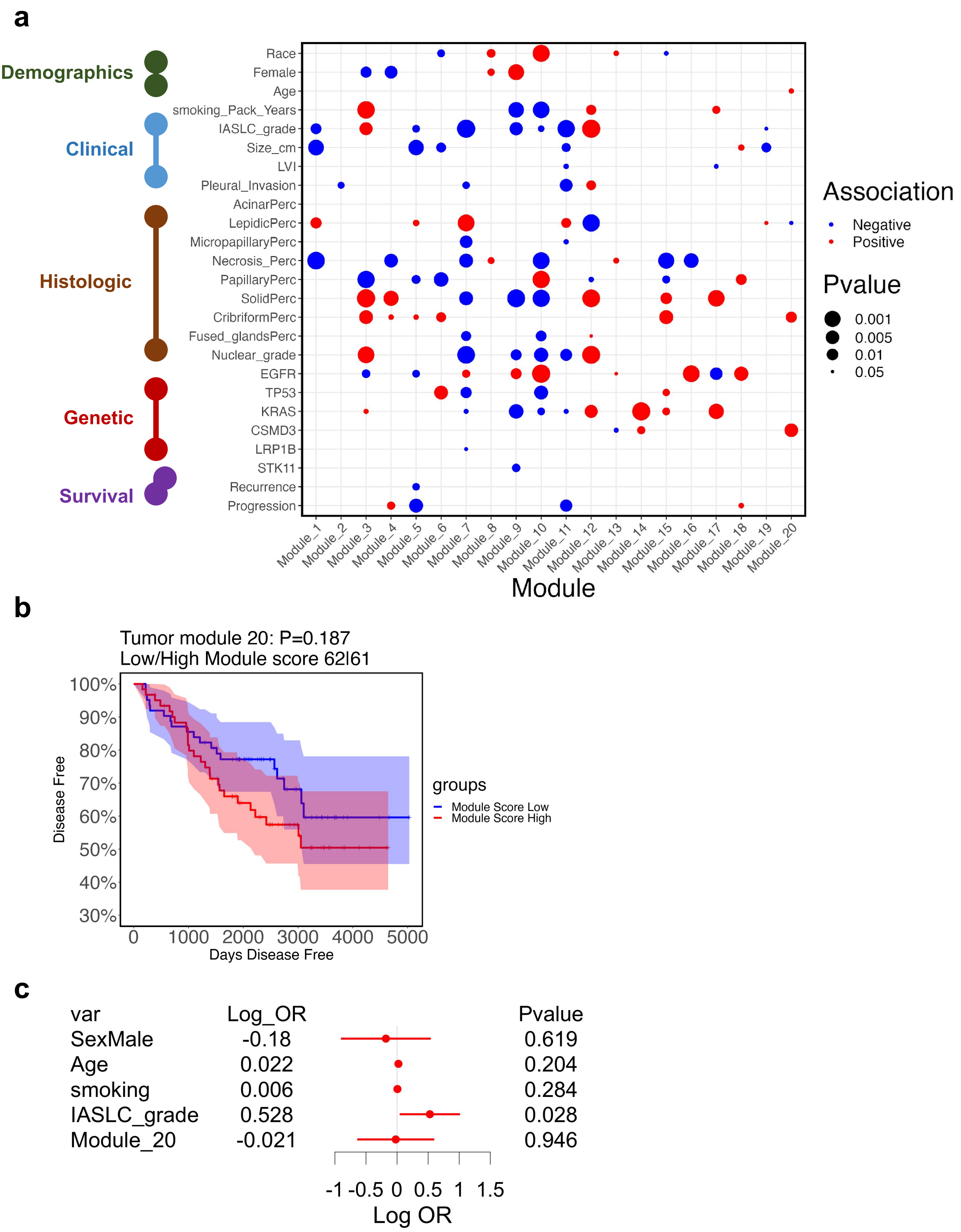
a
Demographics
Clinical
Histologic
Genetic
Survival
Race
Female
Age
smoking_Pack_Years
IASLC_grade
Size_cm
LVI
Pleural_Invasion
AcinarPerc
LepidicPerc
MicropapillaryPerc
Necrosis_Perc
PapillaryPerc
SolidPerc
CribriformPerc
Fused_glandsPerc
Nuclear_grade
EGFR
TP53
KRAS
CSMD3
LRP1B
STK11
Recurrence
Progression
Module_1
Module_2
Module_3
Module_4
Module_5
Module_6
Module_7
Module_8
Module_9
Module_10
Module_11
Module_12
Module_13
Module_14
Module_15
Module_16
Module_17
Module_18
Module_19
Module_20
Module
Association
Negative
Positive
Pvalue
0.001
0.005
0.01
0.05
b
Tumor module 20: P=0.187
Low/High Module score 62|61
100%
90%
80%
70%
60%
50%
40%
30%
Disease Free
0
1000
2000
3000
4000
5000
Days Disease Free
groups
Module Score Low
Module Score High
c
var
Log_OR
Pvalue
SexMale
-0.18
0.619
Age
0.022
0.204
smoking
0.006
0.284
IASLC_grade
0.528
0.028
Module_20
-0.021
0.946
-1
-0.5
0
0.5
1
1.5
Log OR

Supplementary Figure 8: Association of module scores in tumor with different variables

(a) Positive and negative associations of demographic, clinical, histologic, genetic and outcomes with module scores in tumor, pearson and spearman correlation tests were done for continuous and categorical variables separately.

(b) Kaplan-Meier progression-free survival curve for patients with high (n=61) and low (n=62) module 20 scores in tumor, p-value determined by the log-rank test and 95% confidence interval is shown in shaded blue and red.

(c) Multivariate modeling of time-to-progression (n=123), log of odds ratio and data are presented as mean values with 95% confidence intervals. P-values are calculated based on Wald test for each variable.

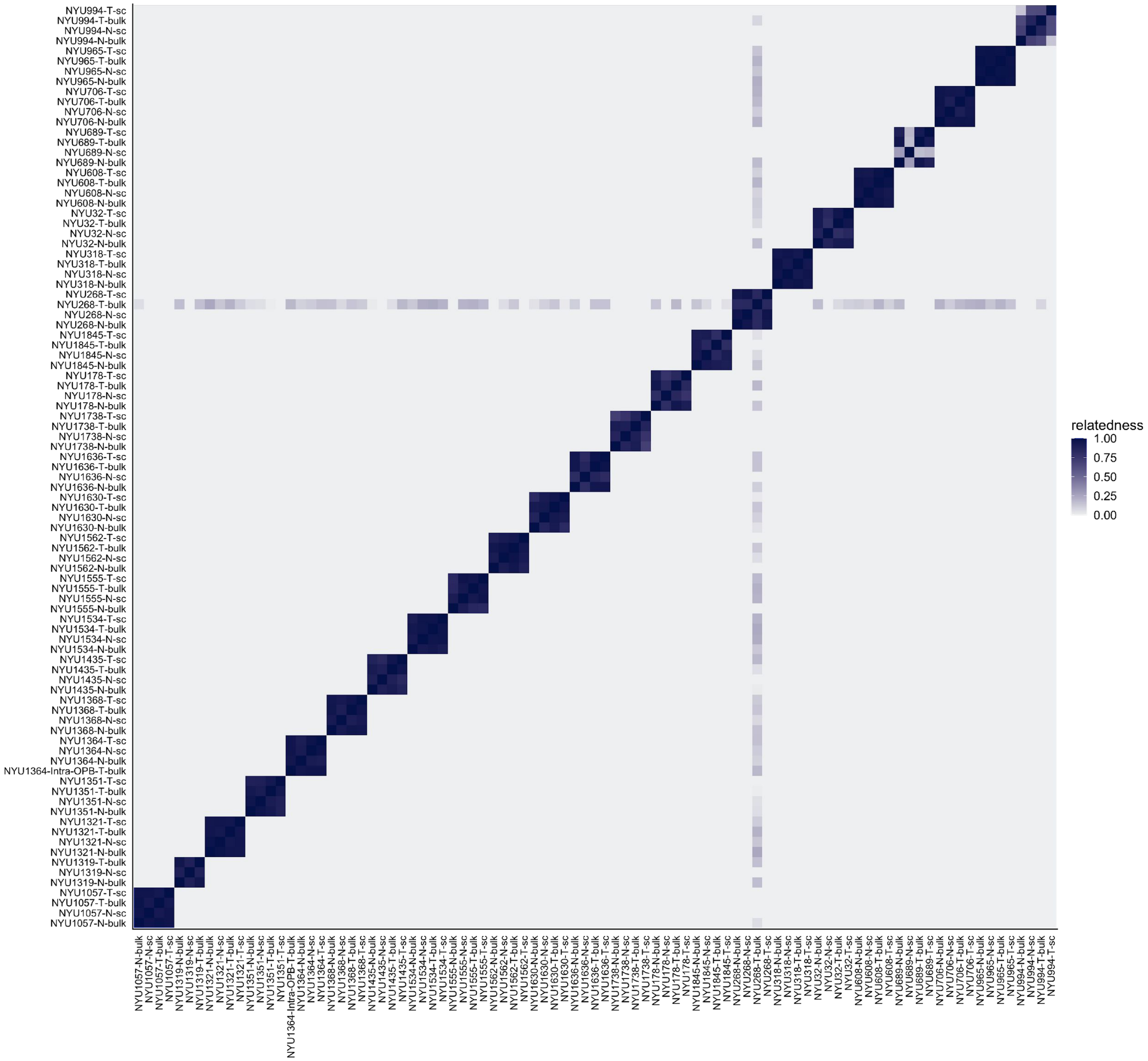
NYU994-T-sc
NYU994-T-bulk
NYU994-N-sc
NYU994-N-bulk
NYU965-T-sc
NYU965-T-bulk
NYU965-N-sc
NYU965-N-bulk
NYU706-T-sc
NYU706-T-bulk
NYU706-N-sc
NYU706-N-bulk
NYU689-T-sc
NYU689-T-bulk
NYU689-N-sc
NYU689-N-bulk
NYU608-T-sc
NYU608-T-bulk
NYU608-N-sc
NYU608-N-bulk
NYU32-T-sc
NYU32-T-bulk
NYU32-N-sc
NYU32-N-bulk
NYU318-T-sc
NYU318-T-bulk
NYU318-N-sc
NYU318-N-bulk
NYU268-T-sc
NYU268-T-bulk
NYU268-N-sc
NYU268-N-bulk
NYU1845-T-sc
NYU1845-T-bulk
NYU1845-N-sc
NYU1845-N-bulk
NYU178-T-sc
NYU178-T-bulk
NYU178-N-sc
NYU178-N-bulk
NYU1738-T-sc
NYU1738-T-bulk
NYU1738-N-sc
NYU1738-N-bulk
NYU1636-T-sc
NYU1636-T-bulk
NYU1636-N-sc
NYU1636-N-bulk
NYU1630-T-sc
NYU1630-T-bulk
NYU1630-N-sc
NYU1630-N-bulk
NYU1562-T-sc
NYU1562-T-bulk
NYU1562-N-sc
NYU1562-N-bulk
NYU1555-T-sc
NYU1555-T-bulk
NYU1555-N-sc
NYU1555-N-bulk
NYU1534-T-sc
NYU1534-T-bulk
NYU1534-N-sc
NYU1534-N-bulk
NYU1435-T-sc
NYU1435-T-bulk
NYU1435-N-sc
NYU1435-N-bulk
NYU1368-T-sc
NYU1368-T-bulk
NYU1368-N-sc
NYU1368-N-bulk
NYU1364-T-sc
NYU1364-N-sc
NYU1364-N-bulk
NYU1364-Intra-OPB-T-bulk
NYU1351-T-sc
NYU1351-T-bulk
NYU1351-N-sc
NYU1351-N-bulk
NYU1321-T-sc
NYU1321-T-bulk
NYU1321-N-sc
NYU1321-N-bulk
NYU1319-T-bulk
NYU1319-N-sc
NYU1319-N-bulk
NYU1057-T-sc
NYU1057-T-bulk
NYU1057-N-sc
NYU1057-N-bulk
NYU1057-N-bulk
NYU1057-N-sc
NYU1057-T-bulk
NYU1057-T-sc
NYU1319-N-bulk
NYU1319-N-sc
NYU1319-T-bulk
NYU1321-N-bulk
NYU1321-N-sc
NYU1321-T-bulk
NYU1321-T-sc
NYU1351-N-bulk
NYU1351-N-sc
NYU1351-T-bulk
NYU1351-T-sc
NYU1364-Intra-OPB-T-bulk
NYU1364-N-bulk
NYU1364-N-sc
NYU1364-T-sc
NYU1368-N-bulk
NYU1368-N-sc
NYU1368-T-bulk
NYU1368-T-sc
NYU1435-N-bulk
NYU1435-N-sc
NYU1435-T-bulk
NYU1435-T-sc
NYU1534-N-bulk
NYU1534-N-sc
NYU1534-T-bulk
NYU1534-T-sc
NYU1555-N-bulk
NYU1555-N-sc
NYU1555-T-bulk
NYU1555-T-sc
NYU1562-N-bulk
NYU1562-N-sc
NYU1562-T-bulk
NYU1562-T-sc
NYU1630-N-bulk
NYU1630-N-sc
NYU1630-T-bulk
NYU1630-T-sc
NYU1636-N-bulk
NYU1636-N-sc
NYU1636-T-bulk
NYU1636-T-sc
NYU1738-N-bulk
NYU1738-N-sc
NYU1738-T-bulk
NYU1738-T-sc
NYU178-N-bulk
NYU178-N-sc
NYU178-T-bulk
NYU178-T-sc
NYU1845-N-bulk
NYU1845-N-sc
NYU1845-T-bulk
NYU1845-T-sc
NYU268-N-bulk
NYU268-N-sc
NYU268-T-bulk
NYU268-T-sc
NYU318-N-bulk
NYU318-N-sc
NYU318-T-bulk
NYU318-T-sc
NYU32-N-bulk
NYU32-N-sc
NYU32-T-bulk
NYU32-T-sc
NYU608-N-bulk
NYU608-N-sc
NYU608-T-bulk
NYU608-T-sc
NYU689-N-bulk
NYU689-N-sc
NYU689-T-bulk
NYU689-T-sc
NYU706-N-bulk
NYU706-N-sc
NYU706-T-bulk
NYU706-T-sc
NYU965-N-bulk
NYU965-N-sc
NYU965-T-bulk
NYU965-T-sc
NYU994-N-bulk
NYU994-N-sc
NYU994-T-bulk
NYU994-T-sc
relatedness
1.00
0.75
0.50
0.25
0.00

Supplementary Figure 9: Genotype-based relatedness of single-nucleus RNA-seq tumor-normal samples to the corresponding bulk RNA-seq samples.

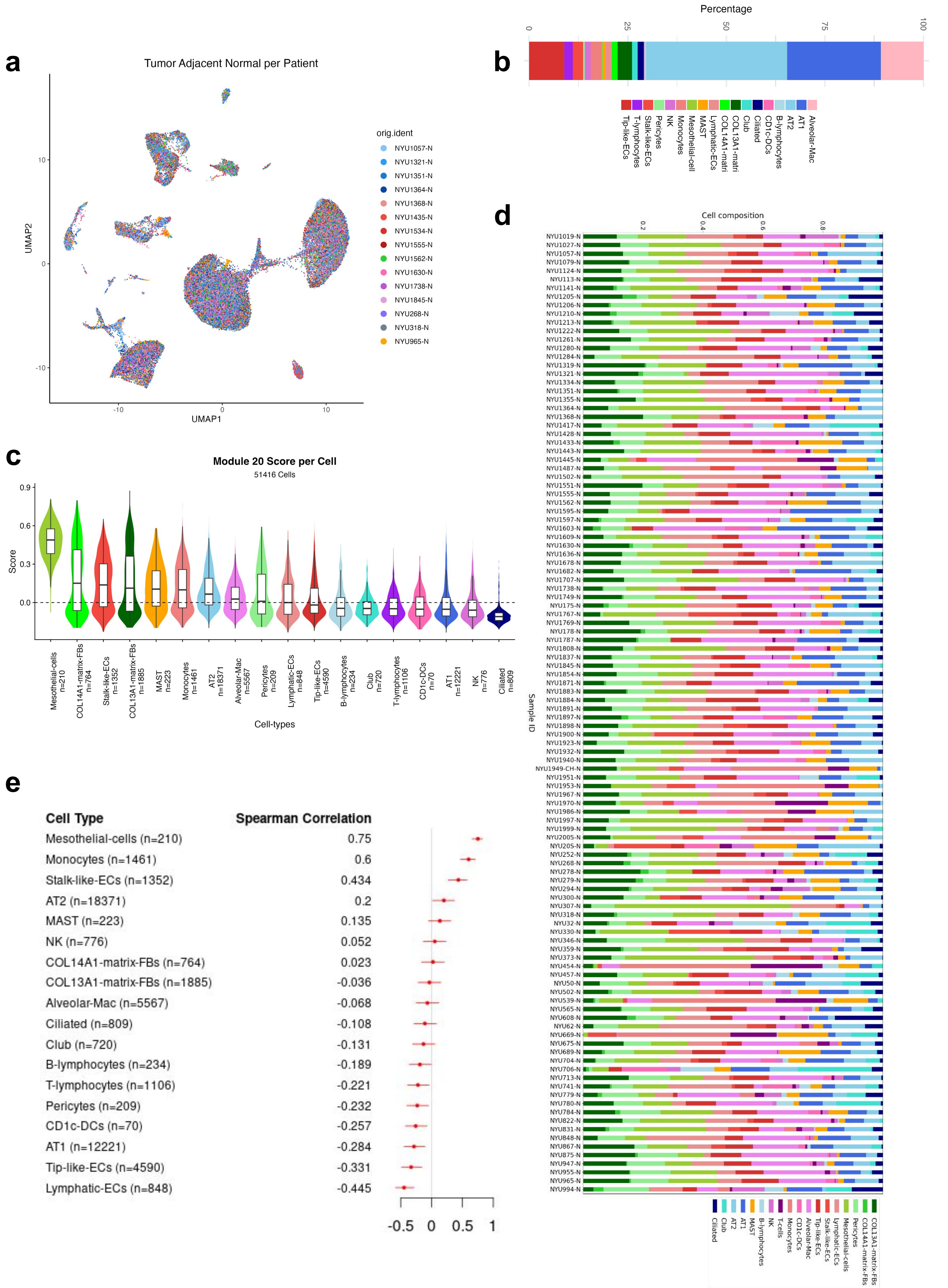
a
Tumor Adjacent Normal per Patient
UMAP1
UMAP2
orig.ident
NYU1057-N
NYU1321-N
NYU1351-N
NYU1364-N
NYU1368-N
NYU1435-N
NYU1534-N
NYU1555-N
NYU1562-N
NYU1630-N
NYU1738-N
NYU1845-N
NYU268-N
NYU318-N
NYU965-N
b
Percentage
Tip-like-ECs
T-lymphocytes
Stalk-like-ECs
Pericytes
NK
Monocytes
Mesothelial-cell
MAST
Lymphatic-ECs
COL14A1-matri
COL13A1-matri
Club
Ciliated
CD1c-DCs
B-lymphocytes
AT2
AT1
Alveolar-Mac
c
Module 20 Score per Cell
51416 Cells
Score
Cell-types
Mesothelial-cells n=210
COL14A1-matrix-FBs n=764
Stalk-like-ECs n=1352
COL13A1-matrix-FBs n=1885
MAST n=223
Monocytes n=1461
AT2 n=18371
Alveolar-Mac n=5567
Pericytes n=209
Lymphatic-ECs n=848
Tip-like-ECs n=4590
B-lymphocytes n=234
Club n=720
T-lymphocytes n=1106
CD1c-DCs n=70
AT1 n=12221
NK n=776
Ciliated n=809
d
Cell composition
Sample ID
Ciliated
Club
AT2
AT1
MAST
B-lymphocytes
NK
T-cells
Monocytes
CD1c-DCs
Alveolar-Mac
Tip-like-ECs
Stalk-like-ECs
Lymphatic-ECs
Mesothelial-cells
Pericytes
COL14A1-matrix-FBs
COL13A1-matrix-FBs
e
Cell Type
Spearman Correlation
Mesothelial-cells (n=210) 0.75
Monocytes (n=1461) 0.6
Stalk-like-ECs (n=1352) 0.434
AT2 (n=18371) 0.2
MAST (n=223) 0.135
NK (n=776) 0.052
COL14A1-matrix-FBs (n=764) 0.023
COL13A1-matrix-FBs (n=1885) -0.036
Alveolar-Mac (n=5567) -0.068
Ciliated (n=809) -0.108
Club (n=720) -0.131
B-lymphocytes (n=234) -0.189
T-lymphocytes (n=1106) -0.221
Pericytes (n=209) -0.232
CD1c-DCs (n=70) -0.257
AT1 (n=12221) -0.284
Tip-like-ECs (n=4590) -0.331
Lymphatic-ECs (n=848) -0.445
-0.5 0 0.5 1

Supplementary Figure 10: Single-nucleus RNA-seq analysis of tumor-adjacent normal samples

(a) UMAP visualization of all 51,416 adjacent normal cells, color-coded by patient.

(b) Cell type abundance in the snRNA-seq dataset.

(c) Per-cell module 20 score distributions grouped by cell type. Boxplots show medians (horizontal line in each box), interquartile ranges (boxes), 1.5 interquartile (whiskers).

(d) Cell type composition barcharts of BayesPrism-deconvoluted bulk RNA-seq tumor-adjacent normal samples.

(e) Spearman correlation of cell type composition with (bulk) module 20 scores across patients. Data are presented as mean values with 95% confidence intervals.

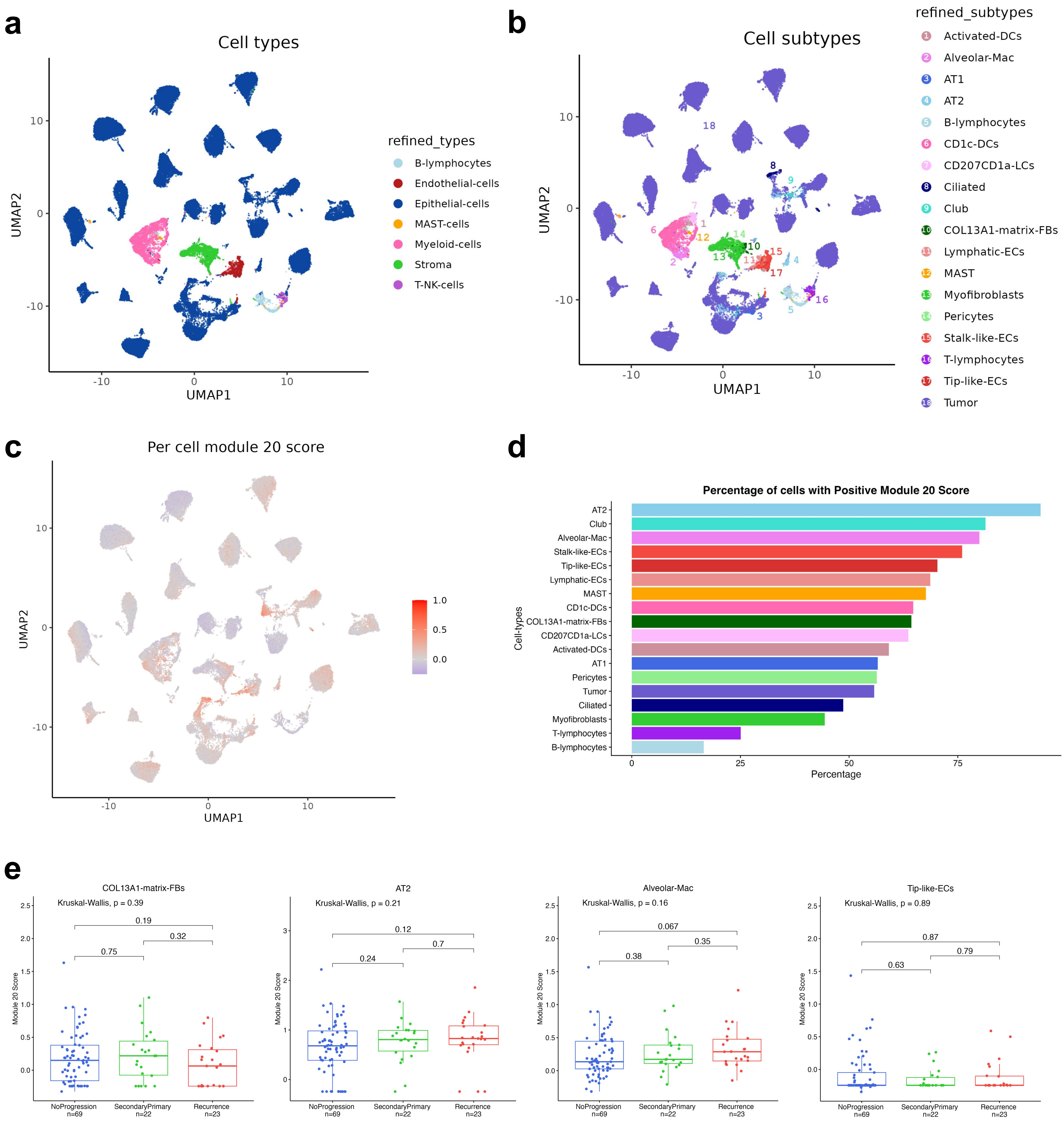
a
Cell types
UMAP2
UMAP1
refined_types
B-lymphocytes
Endothelial-cells
Epithelial-cells
MAST-cells
Myeloid-cells
Stroma
T-NK-cells
b
Cell subtypes
UMAP2
UMAP1
refined_subtypes
1 Activated-DCs
2 Alveolar-Mac
3 AT1
4 AT2
5 B-lymphocytes
6 CD1c-DCs
7 CD207CD1a-LCs
8 Ciliated
9 Club
10 COL13A1-matrix-FBs
11 Lymphatic-ECs
12 MAST
13 Myofibroblasts
14 Pericytes
15 Stalk-like-ECs
16 T-lymphocytes
17 Tip-like-ECs
18 Tumor
c
Per cell module 20 score
UMAP2
UMAP1
d
Percentage of cells with Positive Module 20 Score
Cell-types
Percentage
AT2
Club
Alveolar-Mac
Stalk-like-ECs
Tip-like-ECs
Lymphatic-ECs
MAST
CD1c-DCs
COL13A1-matrix-FBs
CD207CD1a-LCs
Activated-DCs
AT1
Pericytes
Tumor
Ciliated
Myofibroblasts
T-lymphocytes
B-lymphocytes
e
COL13A1-matrix-FBs
Kruskal-Wallis, p = 0.39
AT2
Kruskal-Wallis, p = 0.21
Alveolar-Mac
Kruskal-Wallis, p = 0.16
Tip-like-ECs
Kruskal-Wallis, p = 0.89
Module 20 Score
NoProgression n=69
SecondaryPrimary n=22
Recurrence n=23

Supplementary Figure 11: Single-nucleus RNA-seq analysis of tumor samples

(a) UMAP visualization of all 61,210 nuclei, color-coded based on the broad cell type annotation.

(b) UMAP visualization of all 61,210 nuclei, color-coded based on the cell subtype annotation.

(c) UMAP colored by module 20 score (calculated per nucleus).

(d) Percentage of cells with a positive module 20 score in each cell subtype.

(e) Expression of the module 20 signature in selected cell types across patient groups; statistical significance is calculated using the Mann-Whitney test (two-sided; the Holm method was used to adjust p-values). Boxplots show medians (horizontal line in each box), interquartile ranges (boxes), 1.5 interquartile (whiskers) and each point represents a patient.
