# Supplementary material for: Inflammation in the tumor-adjacent lung as a predictor of clinical outcome in lung adenocarcinoma
Source: Nat Commun. 2023 Nov 8;14:6764. doi: 10.1038/s41467-023-42327-x (PMC10632519; doi:10.1038/s41467-023-42327-x)
Supplement: Supplementary file 2 — Reporting Summary [file 41467_2023_42327_MOESM2_ESM.pdf]

nature portfolio

Corresponding author(s): Aristotelis Tsirigos
Harvey Pass

Last updated by author(s): Aug 31, 2023

Reporting Summary

Nature Portfolio wishes to improve the reproducibility of the work that we publish. This form provides structure for consistency and transparency in reporting. For further information on Nature Portfolio policies, see our Editorial Policies and the Editorial Policy Checklist.

Statistics

For all statistical analyses, confirm that the following items are present in the figure legend, table legend, main text, or Methods section.

n/a	Confirmed	
☐	☒	The exact sample size (n) for each experimental group/condition, given as a discrete number and unit of measurement
☐	☒	A statement on whether measurements were taken from distinct samples or whether the same sample was measured repeatedly
☐	☒	The statistical test(s) used AND whether they are one- or two-sided<br>*Only common tests should be described solely by name; describe more complex techniques in the Methods section.*
☐	☒	A description of all covariates tested
☐	☒	A description of any assumptions or corrections, such as tests of normality and adjustment for multiple comparisons
☐	☒	A full description of the statistical parameters including central tendency (e.g. means) or other basic estimates (e.g. regression coefficient) AND variation (e.g. standard deviation) or associated estimates of uncertainty (e.g. confidence intervals)
☐	☒	For null hypothesis testing, the test statistic (e.g. F, t, r) with confidence intervals, effect sizes, degrees of freedom and P value noted<br>*Give P values as exact values whenever suitable.*
☐	☒	For Bayesian analysis, information on the choice of priors and Markov chain Monte Carlo settings
☒	☐	For hierarchical and complex designs, identification of the appropriate level for tests and full reporting of outcomes
☒	☐	Estimates of effect sizes (e.g. Cohen's d, Pearson's r), indicating how they were calculated

Our web collection on statistics for biologists contains articles on many of the points above.

Software and code

Policy information about availability of computer code

Data collection	Sequencing results were demultiplexed and converted to FASTQ format using Illumina bcl2fastq (v2.0) software.
Data analysis	All software used in this analysis is freely available.<br><br>DNA-sequencing: The DNA fastq files were processed using the Seq-N-Slide (v22.01) pipeline that trims the reads with Trimmomatic (v0.39) and aligns the reads to hg38 using the BWA-MEM (v0.7) algorithm. Following this alignment, Sambamba (v1.0) is utilized to filter out both low-confidence mappings and duplicate DNA reads. Further in the analysis, GATK (v3.8) pinpoints genetic variants while Mutect (v4.1.9) specializes in detecting somatic mutations within cancer samples. Strelka (2.9.10) focuses on identifying genetic variants by comparing matched tumor-normal DNA samples. The identified genetic variants undergo a deeper annotation with ANNOVAR (2017Jul16), offering functional and contextual insights. Furthermore, to validate the detected somatic mutations' relevance in cancer, they are cross-referenced with the COSMIC database (v94 and gnomAD database (v2.1.1).<br><br>RNA-sequencing:The bulk RNA fastq files were processed using the Seq-N-Slide (v22.02) pipeline that uses Trimmomatic (v0.39) and STAR aligner (v2.7.3) . Generating counts for each gene was done with the featureCounts (v1.6.3) and DESeq2 (v1.40.2) was used for differential expression analysis. Somalier (v0.2.18) was used for pairwise correlation. Glmnet (v4.1) was used for elastic net regularization for our logistic regression model. For clustering, the cluster R package (v2.1.4) used the Partitioning Around Medoids (PAM) method, and the uwot R package (v0.1.16) was used to generate UMAP visualizations. Data visualizations were also constructed using the ggplot2 (v3.3.6).<br><br>sn-RNA sequencing: Single-nuclei sequencing reads were trimmed using cutadapt (v4.2). Barcode processing and gene quantification was done by Starsolo (v2.7.3). Seurat (v4.0) was used for QC and Harmony (v0.0.1) was used for batch-correction. scDblFinder (v1.6.0) was used

for doublet removal and SingleR (v1.6.1) was used to annotate the nuclei. InferCNV (v1.11.2) was used to identify tumor cells based on copy number variant scores. BayesPrism (v2.0) was run using default settings for deconvolution of the bulk data.

The github for our code can be found here: https://github.com/ninashenker/LUAD.

For manuscripts utilizing custom algorithms or software that are central to the research but not yet described in published literature, software must be made available to editors and reviewers. We strongly encourage code deposition in a community repository (e.g. GitHub). See the Nature Portfolio guidelines for submitting code & software for further information.

Data

Policy information about availability of data

All manuscripts must include a data availability statement. This statement should provide the following information, where applicable:

- Accession codes, unique identifiers, or web links for publicly available datasets
- A description of any restrictions on data availability
- For clinical datasets or third party data, please ensure that the statement adheres to our policy

The processed DNA-sequencing, RNA sequencing, and single-nucleus data can be found on FigShare (https://doi.org/10.6084/m9.figshare.21331284). The raw snRNA-seq and bulk RNA-seq fastqs were submitted to Gene Expression Omnibus (GEO) repository and can be accessed under GEO accession no. GSE229706. The external TCGA dataset used to compare to ours is available at https://gdc.xenahubs.net. Our data was aligned to the hg38/GRCh38 assembly available at: https://hgdownload.soe.ucsc.edu/goldenPath/hg38/bigZips/hg38.fa.gz. All data generated and supporting the paper are available within this paper.

Human research participants

Policy information about studies involving human research participants and Sex and Gender in Research.

Reporting on sex and gender	In our study, we ensured a balanced representation of participants, with an even distribution based on biological sex, incorporating equal numbers of male and female participants. Additionally, our cohort spanned a wide age range, ensuring robust representation across various age groups. Upon analysis, we found consistent outcomes across all participants. Specifically, neither biological sex nor age exhibited any statistically significant influence on progression outcomes. These findings emphasize that, within the context of our study, other potential factors or variables may be more pivotal in determining progression than age or sex.
Population characteristics	Our study participants were all stage I lung adenocarcinoma patients with matched tumor and tumor-adjacent normal (TAN) tissue samples. Our study participants were of both sexes and ranged in age from 46-89 years. Patients included in the study at no time prior to surgery ever received any treatment for cancer (i.e. radiation, immunotherapy, or chemotherapy).
Recruitment	These were stage I LUAD patients at NYU Langone Health. There are no apparent biases. Patients were not compensated.
Ethics oversight	All patients were resected between 2006 and 2015 after signing informed consent for the New York Langone Health IRB continuously approved protocol i8896 C24 (The NYU Lung Cancer Biomarker Center approved May 6, 2020-April 17, 2024).

Note that full information on the approval of the study protocol must also be provided in the manuscript.

Field-specific reporting

Please select the one below that is the best fit for your research. If you are not sure, read the appropriate sections before making your selection.

[x] Life sciences [ ] Behavioural & social sciences [ ] Ecological, evolutionary & environmental sciences

For a reference copy of the document with all sections, see nature.com/documents/nr-reporting-summary-flat.pdf

Life sciences study design

All studies must disclose on these points even when the disclosure is negative.

Sample size	No power analyses were performed prior to experiments. Single cell datasets were filtered for quality. 123 bulk RNA patients and 33 tumor/normal samples were originally included after QC.
Data exclusions	No data was excluded from the analysis after QC.
Replication	Using public datasets from TCGA, we show that the same inflammatory score can stratify patients in other cancer types.
Randomization	Patients were grouped by recurrence (no recurrence, recurrence) and progression (secondary primary, locoregional, systemic) status.
Blinding	No blinding was involved in this study. Direct classification of each patient and the tumor/normal subtype was required for this study.

Reporting for specific materials, systems and methods

We require information from authors about some types of materials, experimental systems and methods used in many studies. Here, indicate whether each material, system or method listed is relevant to your study. If you are not sure if a list item applies to your research, read the appropriate section before selecting a response.

Materials & experimental systems

n/a	Involved in the study	
☒	☐	Antibodies
☒	☐	Eukaryotic cell lines
☒	☐	Palaeontology and archaeology
☒	☐	Animals and other organisms
☒	☐	Clinical data
☒	☐	Dual use research of concern

Methods

n/a	Involved in the study	
☒	☐	ChIP-seq
☒	☐	Flow cytometry
☒	☐	MRI-based neuroimaging
