# Supplementary material for: Inflammation in the tumor-adjacent lung as a predictor of clinical outcome in lung adenocarcinoma
Source: Nat Commun. 2023 Nov 8;14:6764. doi: 10.1038/s41467-023-42327-x (PMC10632519; doi:10.1038/s41467-023-42327-x)
Supplement: Supplementary file 3 — Description of Additional Supplementary Files [file 41467_2023_42327_MOESM3_ESM.pdf]

Description of Additional Supplementary Files

Supplementary Data 1
Description: Cohort characteristics

Supplementary Data 2
Description: DNA sequencing quality assessment

Supplementary Data 3
Description: RNA sequencing quality assessment

Supplementary Data 4
Description: DNA sequencing mutation calls

Supplementary Data 5
Description: Module gene set enrichment analysis (KEGG pathways, Gene Ontology and HALLMARKS)

Supplementary Data 6
Description: Differential gene expression analysis
